# Potato Annexin STANN1 Promotes Drought Tolerance and Mitigates Light Stress in Transgenic *Solanum tuberosum* L. Plants

**DOI:** 10.1371/journal.pone.0132683

**Published:** 2015-07-14

**Authors:** Michal Szalonek, Barbara Sierpien, Wojciech Rymaszewski, Katarzyna Gieczewska, Maciej Garstka, Malgorzata Lichocka, Laszlo Sass, Kenny Paul, Imre Vass, Radomira Vankova, Peter Dobrev, Pawel Szczesny, Waldemar Marczewski, Dominika Krusiewicz, Danuta Strzelczyk-Zyta, Jacek Hennig, Dorota Konopka-Postupolska

**Affiliations:** 1 Plant Pathogenesis Lab, Institute of Biochemistry and Biophysics Polish Academy of Science, Warsaw, Poland; 2 Department of Metabolic Regulation, University of Warsaw, Warsaw, Poland; 3 Laboratory of Molecular Stress and Photobiology, Biological Research Centre of the Hungarian Academy of Sciences, Szeged, Hungary; 4 Laboratory of Hormonal Regulations in Plants, Institute of Experimental Botany AS CR, Praha, Czech Republic; 5 Department of Potato Genetics and Parental Lines, Plant Breeding and Acclimatization Institute—National Research Institute, Mlochow, Poland; Chinese Academy of Sciences, CHINA

## Abstract

Annexins are a family of calcium- and membrane-binding proteins that are important for plant tolerance to adverse environmental conditions. Annexins function to counteract oxidative stress, maintain cell redox homeostasis, and enhance drought tolerance. In the present study, an endogenous annexin, STANN1, was overexpressed to determine whether crop yields could be improved in potato (*Solanum tuberosum* L.) during drought. Nine potential potato annexins were identified and their expression characterized in response to drought treatment. *STANN1* mRNA was constitutively expressed at a high level and drought treatment strongly increased transcription levels. Therefore, *STANN1* was selected for overexpression analysis. Under drought conditions, transgenic potato plants ectopically expressing STANN1 were more tolerant to water deficit in the root zone, preserved more water in green tissues, maintained chloroplast functions, and had higher accumulation of chlorophyll *b* and xanthophylls (especially zeaxanthin) than wild type (WT). Drought-induced reductions in the maximum efficiency and the electron transport rate of photosystem II (PSII), as well as the quantum yield of photosynthesis, were less pronounced in transgenic plants overexpressing STANN1 than in the WT. This conferred more efficient non-photochemical energy dissipation in the outer antennae of PSII and probably more efficient protection of reaction centers against photooxidative damage in transgenic plants under drought conditions. Consequently, these plants were able to maintain effective photosynthesis during drought, which resulted in greater productivity than WT plants despite water scarcity. Although the mechanisms underlying this stress protection are not yet clear, annexin-mediated photoprotection is probably linked to protection against light-induced oxidative stress.

## Introduction

Plants have developed passive and active strategies to survive environmental stresses such as drought, salinity, chilling, heat shock, heavy metals, UV radiation, ozone, mechanical stress, nutrient deficiency, hypoxia, and biotic stress [1]. Several stress-response genes have already been targets for bioengineering studies to improve plant stress tolerance [2]. However, ectopic expression of stress-inducible genes often results in developmental aberrations (e.g., stunted growth and irregular leaves) or reduced crop yields under non-stress conditions due to non-specific induction of programmed cell death (PCD) and/or premature senescence [3]. Current knowledge of stress-responsive pathways is based primarily on results obtained by imposing each stress individually, whereas plants in natural settings are generally challenged with multiple concurrent stresses, and the resultant signaling pathways may be superimposed and/or induce/antagonize each another [4–6]. New approaches to bioengineering stress tolerance in crop plants are needed to achieve sustainable improvements in crop biomass production [2].

Recent work shows that changes in redox poise can regulate plant cell function [7–9] by acting as cellular signals [10]. In light, the predominant location of reactive oxygen species (ROS) production is in chloroplasts [11]. The two main ROS sources there are the light-driven photosynthetic electron transport chains (PETCs) of photosystem I (PSI) and photosystem II (PSII). Environmental stress can trigger an imbalance in redox homeostasis and affect chloroplast metabolism [12]. Abiotic stresses reduce CO_2_ assimilation, which results in over-reduction of the PETC and utilization of oxygen as an alternative acceptor for excess electrons [13]. Changes in chloroplast redox poise activate secondary ROS-producing sources, such as membrane NADPH oxidase complex {respiratory burst oxidase homologs (RBOHs)} [14] or photorespiration [15].

Annexins are a multigene, evolutionarily conserved family of calcium- and phospholipid-binding proteins [16] that is present in all eukaryotes. They have a highly conserved tertiary structure defined by the presence in the molecule of four (or eight) approximately 70 amino acid motifs, each consisting of five α-helices. The contribution of annexins to plant cell adaptation to adverse environmental conditions is well documented [16–20]. However, an understanding of the primary physiological functions of plant annexins remains elusive. Initially, based on their prototypical characteristics, annexins were thought to participate primarily in membrane-related events, such as cellular transport, membrane-cytoskeleton interactions, or endo-/exocytosis [16, 20]. Later, it became clear that their cellular functions go far beyond this and include regulation of cellular redox poise and modulation of calcium transients upon stress.

Annexin 1 was identified in a genome-wide search of *Arabidopsis thaliana* (Arabidopsis) sequences capable of rescuing *Escherichia coli* ΔoxyR growth on high H_2_O_2_ concentrations [21]. Further experiments showed that Arabidopsis annexin 1 (ATANN1) is an element of the ROS signaling network in Arabidopsis. Deletion of functional ATANN1 reduced expression of glutathione-*S*-transferase Tau 1 (GSTU1) in seedlings after H_2_O_2_ treatment [22]. It is well accepted that upon salinity stress, ROS trigger increases in Ca^2+^, and ATANN1 was proposed to mediate calcium conductance activated by NADPH oxidases in root epidermal cells [22]. These data suggest that ATANN1 can function at a cross-road of calcium and ROS signaling [23, 24]. It is still an open question if other proteins from this family can function in a similar way.

Subsequent analyses confirmed that not only ATANN1, but also *Brassica juncea* annexins BJANN1 and BJANN3, and *Nelumbo nucifera* annexin NNANN1 ameliorated oxidative stress in homologous or heterologous cells and improved stress tolerance of tobacco, cotton, and Arabidopsis [23, 25–30]. In some cases, overexpression of annexins resulted in multi-stress tolerance. Transgenic tobacco plants expressing BJANN1 exhibited enhanced resistance to different abiotic stresses and infection with *Phytophthora parasitica* var. *nicotianae*, the latter possibly due to constitutively increased expression of several pathogenesis-related proteins [25]. NNANN1 overexpression in Arabidopsis conferred enhanced tolerance to heat and oxidative stress [27]. These studies generally used seedlings or leaf discs subjected to short-term stress treatments (in hours). There is a lack of information regarding annexin function in cell physiology, hormonal homeostasis, and metabolism during long-term exposure to environmental stress.

Potato (*Solanum tuberosum* L.) is one the most important vegetable crops. Its global annual production in 2010 exceeded 300 million tons (FAOStat). Potato plants are highly efficient in terms of water usage (http://www.fao.org/potato-2008/en/potato/water.html), and produce more food per water unit than any other crop [31]. Therefore, potato could be a promising alternative to cereal crops. Modern potato cultivars are susceptible to drought, which is defined as a shortage of water in the root zone [32]. Water deficit affects nearly all stages of potato development, and negatively impacts tuber numbers and quality (crop yield) [33, 34]. Only a few attempts to engineer potato drought tolerance have been reported (reviewed in [35]). These studies had limited success because most transgenic plants did not exhibit good performance and productivity under non-stress and stress conditions. Potato annexin has not been considered for bioengineering applications; however, new proteomics research showed that STANN1 could be a candidate gene to improve stress tolerance. STANN1 was differentially expressed in potato tubers in response to wounding [36, 37], bruising (personal observation; [38]), osmotic stress and salinity [39], and was differentially expressed in potato aerial parts in response to osmotic stress and salinity [40]. We overexpressed potato annexin STANN1 and observed the effects on plant biochemistry and physiology during drought.

First, we investigated if increased expression of ATANN1 affected potato drought tolerance. We used the *S*. *tuberosum* genome to identify all potato annexins, and analyzed potential involvement in drought responses using semi-quantitative RT-PCT. Then, we characterized photosynthetic performance in transgenic plants overexpressing ATANN1 during prolonged water deficit around the root zone. One of the plant strategies to cope with environmental stresses is premature induction of the senescence program [41]; therefore, we analyzed the influence of STANN1 on long-lasting changes in hormonal homeostasis during drought. We also investigated possible annexin functions in modulating redox signaling, and assessed changes in drought stress responses. Our working hypothesis was that annexin modulated plant stress responses by increasing the cytosolic antioxidant buffering capacity in transgenic plants. Studies on Arabidopsis ecotypes indicate that *ATANN1* mRNA levels differ in ecotypes adapted to very different local climatic condition (TAIR and our non-published data). In potato tubers, STANN1 levels did not differ in proteomes from different genetic backgrounds [42]. Further experiments are necessary to elucidate if drought-tolerant potato landraces and cultivars could be generated by enhancing the level of annexin expression.

## Materials and Methods

### Generation of transgenic plants, transformation and growth conditions

*S*. *tuberosum* cultivar Sante (WT), medium-tolerant to drought, was used for transformation experiment (http://www.europotato.org). The *STANN1* cDNA sequence without the stop codon (957 bp; Acc. No. PGSC0003DMG400017714) was fused at the 3′ end to a 6×His-tag sequence and inserted into the XbaI restriction site of pROK2 [43] between cauliflower mosaic virus 35S promoter and nopaline synthase (Nos) terminator sequences (Fig A in S1 File). This construct was used for *Agrobacterium tumefaciens*-mediated transformation of WT potato plants according to a previously published method [44]. Regenerated transgenic plants were transplanted into separate glass tubes filled with 10 mL of Murashige & Skoog solid medium supplemented with 50 μg/mL kanamycin. The presence of the transgene cassette was verified with genomic PCR (data not shown). Expression of recombinant STANN1_6×His protein was confirmed by purification from leaves of WT and F1 transgenic plants (lines S-2, S-3, S-7, S-83, S91, S-97, and S-123) by Ni-NTA chromatography and detection with anti-HisTag primary antibody (Sigma-Aldrich). Recombinant ATANN1_6×His protein produced by bacterial overexpression was used as a positive control (WT protein extract) (Fig A in S1 File).

Potato WT plants or transgenic lines in the “Sante” background (S-2 and S-7) were used for further experiments. Plants were cultivated in a growth chamber (or an air-conditioned greenhouse when indicated) under standard conditions (21±2°C; 16 h/8 h day/night; light intensity 110 to 130 PPFD (photosynthetic photon flux densities); 60–80% relative humidity.

### Water stress

*S*. *tuberosum* plantlets sprouted from tubers were grown in plastic pots filled with 1 kg of sterilized soil (mixture of peat and sand, pH 5.5; prepared by the Plant Breeding and Acclimatization Institute) for 160–170 days. The field capacity (FC) was determined gravimetrically (g of water per g of soil). Pots were weighed every 2–3 days and the volume of water necessary to maintain the indicated FC was calculated individually for each plant. For well-watered control plants, FC was maintained at 65% (−0.8 MPa) for the whole experiment. Experimental drought was imposed after 8–10 weeks of growth (tuber initiation) (Fig B in S1 File). Irrigation was decreased over 10 days to gradually reduce the FC to ~25% FC (−2.0 MPa) and was then maintained at this level until the end of the water deficit period. Irrigation was subsequently resumed with full soil saturation (rewatering). To estimate the impact of drought on potato productivity, plants were cultivated for an additional 11–12 weeks after rewatering (FC 65%) until physiological maturity. An exemplary schedule of FC changes is shown in Fig B in S1 File. Samples were collected at the beginning of the water deficit period (D0), and (depending on experiment) at different days of drought, i.e. 3rd (D3), 4th (D4), 6th (D6), 10th (D10), and 14th (D14), and at the first (RW1) and third (RW3) days after rewatering.

### Identification of potato annexins

Annexins were identified in silico by searching for the endonexin domain (PFAM definition, PF00191, 66 aa) in six translation frames of the heterozygous diploid potato breeding line, *S*. *tuberosum* L. group Tuberosum RH89-039-16 genome using the HMMSearch program from the HMMER3 package. According to PFAM >93% of proteins from this family contained at least three consecutive repeats of the endonexin domain. By searching with a single repeat, the probability of missing a complete protein due to below-threshold partial hits or incorrectly defined intron-exon boundaries was minimized. Only hits with an E-value ≤0.001 were considered. To verify the presence and sequence of the predicted annexins in WT potato, genome primer sets were designed that corresponded to the 5′ (F) and 3′ (R) ends of the predicted open reading frames (ORFs, Table A in S2 File). Expression of putative annexin genes was verified using RT-PCR. Briefly, total RNA was isolated from WT leaves and reverse transcribed using RevertAid Reverse Transcriptase (Thermo Scientific, Lithuania) with poly(T)_12–18_ primer. Annexins were amplified from cDNA using Phusion High-Fidelity DNA Polymerase (Thermo Scientific, Lithuania). PCR products were cloned with pJET Cloning Kit (Thermo Scientific, Lithuania) and their compliance with the predicted sequences was verified.

### Semi-quantitative expression of annexins and stress-regulated genes

Gene expression was profiled over 14 days of drought in WT potatoes grown as described above. Samples were taken from the first fully-developed composite leaf at the top of the plant. For each time point, single leaf discs from four independent plants were collected, flash-frozen in liquid nitrogen, and stored at −80°C until use. Total RNA was isolated with Trizol (Invitrogen, Scottland, UK). Reverse transcription was performed as described above. Taq DNA Polymerase (Thermo Scientific, Lithuania) was used to amplify specific sequences from cDNA. Genes for semi-quantitative analysis were selected from PGSC_DM_v3.4_pep_fasta, which contains a database of potato virtual translation products predicted according to similarity to annotated Arabidopsis genes. Specific primer sets for expression analysis were designed using PrimerSelect, Laser Gene10.0 DNASTAR (USA) (Table B in S2 File). The obtained sq-RT-PCR products were subjected by agarose gel electrophoresis, stained with ethidium bromide, and quantitated by densitometry using MultiGaugeV3.0 (Fuji) software. Expression was normalized with respect to the expression of potato elongation factor 1 alpha mRNA (EF1a; PGSC0003DMT400050664; [45]). Each single experiment included four biological replicates, which were quantitated in three technical replicates. Experiments were repeated three times for each primer set and template.

### Relative water content

Relative water content (RWC) was determined as described previously [46] with the slight modification. For full saturation {equivalent to turgor weight (TW)} leaves were incubated in distilled water for 4 h instead of overnight. Experiments were performed three times on at least five biological replicates for each genotype.

### Extraction and determination of plant hormones

Leaf samples of ~0.5 g (without the main vein) from 8–10-week plants subjected to drought (as described above) were collected, immediately frozen in liquid nitrogen, and kept at −80°C until use. Samples were taken from the first fully-developed composite leaf at the top of the plant at the indicated time points. Sampling was performed 4 h after the start of daily illumination. Three independent biological replicates were examined. Purification and analysis were performed as described previously [47, 48]. Briefly, leaf samples were homogenized and extracted with methanol/water/formic acid (15/4/1, v/v/v) and the following labeled internal standards (10 pmol per sample) were added: ^2^H_6_-ABA, ^2^H_5_-*trans*Z, ^2^H_5_-*trans*ZR, ^2^H_5_-*trans*Z7G, ^2^H_5_-*trans*Z9G, ^2^H_5_-*trans*ZOG, ^2^H_5_-*trans*ZROG, ^2^H_5_-*trans*ZRMP, ^2^H_3_-DHZ, ^2^H_3_-DHZR, ^2^H_3_-DHZ9G, ^2^H_6_-iP, ^2^H_6_-iPR, ^2^H_6_-iP7G, ^2^H_6_-iP9G, and ^2^H_6_-iPRMP (Olchemim, Czech Republic). Extracts were purified using a SPE-C18 column (SepPak-C18, Waters), and separated on a reverse phase-cation exchange SPE column (Oasis-MCX, Waters). The first hormone fraction, containing abscisic acid (ABA) and its metabolites, was eluted with methanol and the second fraction (containing CK metabolites)] was eluted with 0.35 M NH_4_OH in 70% methanol. Both fractions were separated by HPLC (Ultimate 3000, Dionex, Switzerland) and the hormones were quantified using a hybrid triple quadruple/linear ion trap mass spectrometer (3200 Q TRAP, Applied Biosystems, USA).

### Gas exchange and chlorophyll fluorescence measurements

Gas exchange and net photosynthesis were analyzed with a Portable Handheld Photosynthesis System CID 340 device (CID Bio-Science, Camas, WA, USA) according to the manufacturer’s instructions. The maximum quantum efficiency of photosynthesis (Fv/Fm) and the effective quantum yield of PSII {Y(II)} were determined with CID 340 (CID Inc., USA) with a CI-510CF Chl fluorescence module and a CI-310LA light attachment (CID Bio-Science) providing actinic light. Measurements were performed 5 h after turning on the light, if not indicated otherwise, on the upper five fully-expanded unwrinkled leaves. Five plants were analyzed per time point. For maximal fluorescence (Fm) determination, plants were dark-adapted for 30 min (so all PSII reaction centers were closed) and then stimulated with saturating pulses of light (0.8 sec, 3,000 PPFD). The minimal fluorescence (Fo) with all PSII reaction centers opened was measured with modulated light of 0.25 PPFD. Fv was calculated from the equation Fv = Fm-Fo. Y(II) was calculated using the equation Y(II) = (Fms-Fs)/Fms. The maximal fluorescence under light (Fms) was determined by allowing plants to adapt to light for 20 min and measuring the steady-state of chlorophyll (Chl) fluorescence (Fs). Next, a saturating pulse (0.8 sec, 3,000 PPFD) was applied and Fms was determined.

Gross non-photochemical quenching (NPQ) was estimated with a Dual Pulse Amplitude Modulation device, PAM-100 (Walz, Germany). For a single time point, six composite leaves from three to five control plants were analyzed. NPQ was calculated as (Fm − Fms)/Fms, where Fm represents the fluorescence of a dark-adapted sample and Fms represents a fluorescence of the illuminated sample. Plants were dark-adapted for ~20 min and kinetics were measured after repeated light pulses of 94 PPFD for 300 sec. Leaves were subsequently relaxed in darkness for 240 sec and fluorescence while continuously measuring and recording fluorescence.

### Non-polar lipids extraction and carotenoids (Car)/Chl determination

Plant material was collected from 8–10-week-old plants exposed to drought. Samples were collected 4 h after switching on the light at D0, D6, D14, and RW3. One leaf disc (11 mm diameter) was taken from the third, fourth, and fifth fully-expanded composite leaves, and total six discs harvested from two plants were combined as a single sample. Non-polar lipids were extracted at 4°C. Plant material after shredding in cooled mortar was transferred to a 15 mL Pyrex tube. After the addition of 3 mL acetone-methanol (8:2 v/v), the sample was perfused with argon and mixed vigorously by vortexing for 2 min. For the second and third extractions, hexane (9 mL) was added and the sample was again perfused with argon before capping and shaking in a reciprocating shaker (PROMAX 2020, Heidolph, Germany) for 30 min in the dark. After shaking, the sample was incubated without agitation for 5 min to allow phase separation. The upper hexane phase was collected by aspiration and transferred to a 100 mL Erlenmeyer flask, perfused with argon, capped and stored in the dark in 4°C. In the second extraction stage, 2 mL of propanol was used in addition to hexane, and perfusion, shaking and phase collection were repeated as before. After removal of hexane, the polar phase was centrifuged for 15 min at 4500 rpm. The supernatant was combined with the two hexane phases, perfused with argon, and filtered through a Milipore syringe filter unit Millex-CV13 Filter Unit (0.22 μm). The combined hexan phases were then transferred to room temperature, evaporated to dryness under argon, and dissolved in 1 mL methanol-propanol-hexane 6:1:3 (v/v/v). Dissolved samples were transferred to 2 mL glass vials, perfused with argon, capped, and stored at −80°C.

Non-polar lipids were analyzed by injecting 5 μL of sample extract onto an ACQUITY UPLC HSS T3 1.0×150 mm 1.8 μm column and eluted with a gradient of solvent A {water and methanol (1:9, v/v)} and solvent B {methanol:isopropanol: hexane (2:1:1, v/v/v)}, with a total of 210 min to transition from solvent A to B. Separation was monitored in the 300–750 nm range with a photodiode array detector. A single chromatogram at 436 nm was extracted, exported in ASCII format, and used for peak area integration analysis with GRAMS/AI software (Thermo Electro Corp, Finland).

Chl*a* and Chl*b* contents were estimated by recording the absorbance of the aforementioned extract at 663, 652, and 645 nm (Cary 50 Bio UV/VIS spectrophotometer, Varian, Australia) as described previously [49].

### ROS levels and lipid peroxidation during high light stress

One leaf disc (11 mm diameter) was taken from the third, fourth, and fifth fully-expanded leaves of potato plants, and for a total of six discs three discs were harvested from each of two plants were combined as a single sample. Immediately after harvesting, samples were vacuum-infiltrated with methyl viologen (MeV) at the indicated concentrations and then incubated in the dark for 1 h under normal irradiance (150 PPFD). Images were obtained after 30 h of incubation.

A similar procedure was used for ROS quantification, with the exception that a single MeV concentration (50 μM) was used and samples were exposed to high irradiance (850 PPFD), well in excess of the levels that saturate photosynthesis in Arabidopsis (high light stress, HL). Samples were collected at the indicated time points. Superoxide anion (O_2_^•−^) content was determined using a colorimetric nitro blue tertrazolium (NBT) assay as described previously [50]. Hydrogen peroxide (H_2_O_2_), was detected with diaminobenzidine tetrahydrochloride (DAB) and quantified by counting pixels on scanned images using ImageJ software [51]. Lipid peroxidation was estimated spectrophotometrically with thiobarbituric acid (TBA) [52].

### Transient expression of STANN1_GFP in *Nicotiana benthamiana*

The *STANN1* sequence (without the stop codon) was introduced between NcoI and BcuI restriction sites at the 5′-end of the monomeric GFP (mGFP) coding sequence in pCAMBIA1302. Intact *N*. *benthamiana* leaves were infiltrated with *A*. *tumefaciens* GV3101 transformed with empty pCAMBIA1302 expressing mGFP or pCAMBIA1302 expressing STANN1_mGFP as described previously [53]. After 3 days, 1 cm diameter leaf discs were excised and incubated with 50 μM MeV for 1 hh in darkness, and then incubated for 4 h in high light (850 PPFD). Fluorescence was immediately observed using a Nikon Eclipse TE2000-E inverted C1 confocal laser scanning microscope equipped with a 40× Plan Fluore oil immersion objective (numerical aperture, 1.30). mGFP and chloroplast autofluorescence were excited with a solid-state Coherent Sapphire 488 nm laser and detected using 515/30 band pass and 610 long pass emission filters, respectively. All samples were analyzed in triplicate. Three independent experiments were performed.

### Statistical analyses

Data were analyzed using two-way ANOVA with Duncan’s Multiple Range Test (DMRT) (for yield) and MANOVA regression models (for other experiments). Multiple comparisons between means were performed with a HSD Tukey test with a confidence limit of 95%.

## Results

### Identification of potato annexin genes

**Fig 1:**
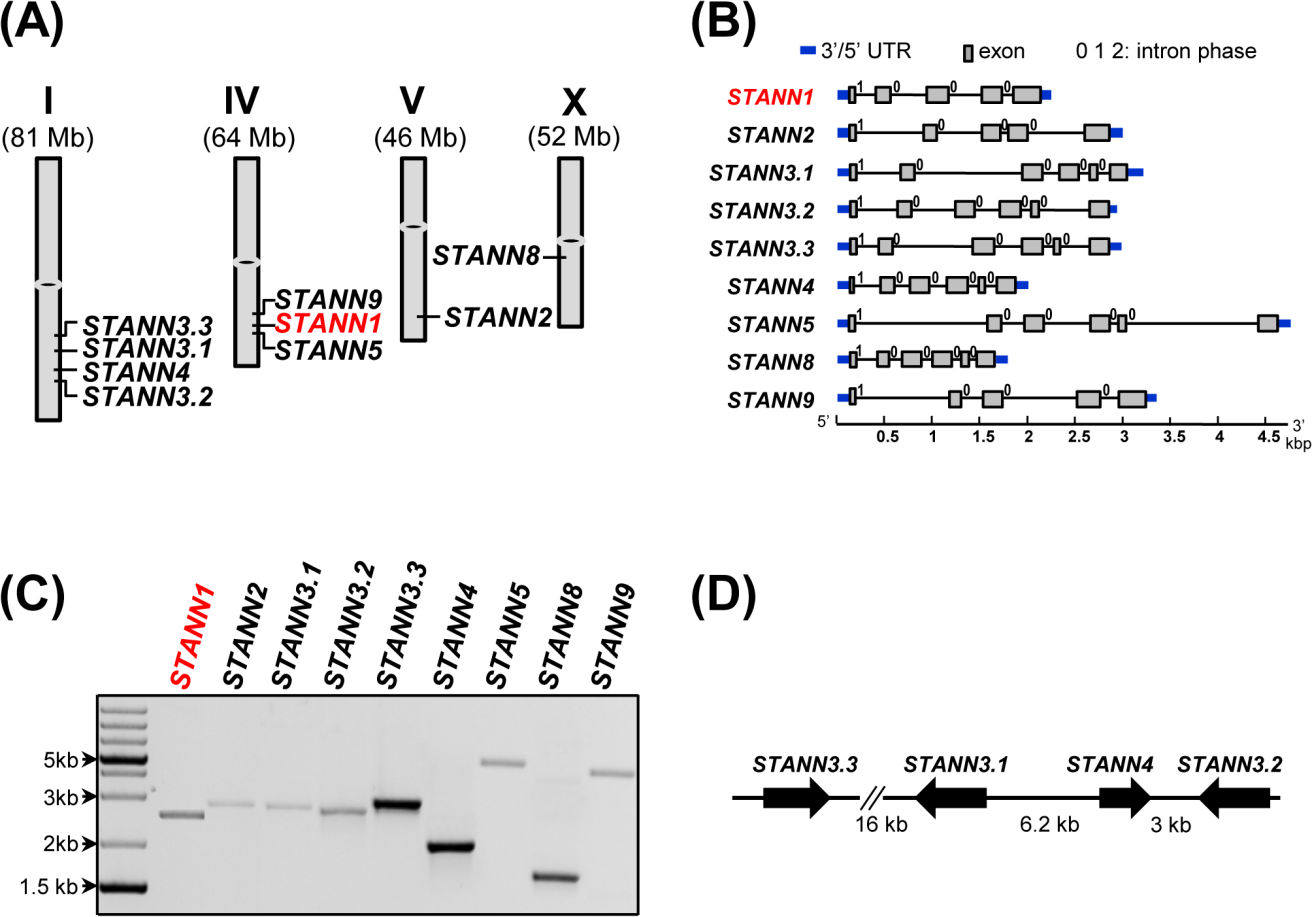
Annexin genes in potato genome. (A) Localization of annexin genes on potato chromosomes. The Roman numerals at the top denote the chromosome, digits in brackets indicate chromosome size. (B) Intron-exon organization of potato annexin genes. (C) Genomic PCR confirming the presence of predicted annexin genes in WT potato. Specific primers anneal to the 5’- and 3’- ends of coding sequence of certain annexin gene, hence the length of the resulting PCR product is a sum of the respective coding sequence with introns. (D) Schematic arrangement of *STANN3*.*1*, *STANN3*.*2*, *STANN3*.*3* and *STANN4* on chromosome I.

Genome-wide examination of the potato sequence database for annexins revealed the presence of 11 DNA segments encoding putative proteins displaying substantial similarity to previously characterized plant annexins. Two of these sequences were classified as pseudogenes due to several defects and a lack of continuity in any of the six ORFs. The remaining nine genes were located on chromosomes I, IV, V, and X and each encoded 5–6 exons (Fig 1A). The positions and phases of introns in the putative potato annexin genes were consistent with those reported for rice annexins [54] (Fig 1B). The putative annexin sequences in the *S*. *tuberosum* genome were verified using genomic PCR (Fig 1C), and the lengths of the amplified genomic products were as expected (Table A in S2 File). The degree of nucleotide sequence identity between the putative potato annexins was 41–92%. Sequences identified by bioinformatics approaches were confirmed experimentally. Reverse transcription polymerase chain reaction indicated that all nine genes were expressed in different potato organs (data not shown).

Multiple alignment of the putative potato annexin amino acid sequences with Arabidopsis annexins revealed that all but one of the potato annexins had Arabidopsis homologs (data not shown). The newly-identified potato genes were named accordingly as *STANN1*, *STANN2*, *STANN3*.*1*, *STANN3*.*2*, *STANN3*.*3*, *STANN4*, *STANN5*, *STANN8*, and *STANN9* (data not shown). The potato annexins formed a functionally diverse protein family that was differentially expressed in different plant organs (data not shown). The most striking genomic feature of the potato annexin family was triplication of the annexin 3 gene on chromosome 1 (Fig 1D). In addition, in an arrangement resembling that in Arabidopsis, potato *STANN3*.*1* and *STANN4* were adjacently localized and divergently transcribed, possibly from a shared promoter. The Inparanoid database groups all annexins in the same in-paralog cluster; however, we suspect that the annexin 3 variants (*STANN3*.*1*, *STANN3*.*2*, and *STANN3*.*3*) are within-species out-paralogs. Two duplications (ancestral gene → *STANN3*.*1* and the ancestor of *STANN3*.*2*; then the ancestor of *STANN3*.*2* → *STANN3*.*2* and *STANN3*.*3*) appear to have occurred prior to potato and tomato speciation, as *S*. *lycopersicum* contains two orthologs of the annexin STANN4 and STANN3.1. In turn, *STANN4* and *STANN3*.*1–3*.*3* are out-paralogs, as *STANN4* is moderately related to all *STANN3* variants but shares high sequence similarity with other annexins from *S*. *lycopersicum* or Arabidopsis. Multiplication of DNA segments within this region of chromosome 1 during *Solanaceae* evolution apparently took place independently at least twice. In tomato chromosome 1, the entire dyad of *SLANN3*/S*LANN4* was duplicated [55] and gave rise to a tetrad located within a short segment of DNA (21,145 bp) that was not interspersed with other genes. This region of chromosome 1 represents a “hot-spot” in the *Solanaceae* family where duplications of a single gene or gene cluster occurred.

### Characteristic of potato annexin proteins

Newly-identified potato annexins had similar predicted molecular masses of 34–37 kDa and diverse isoelectric points (5.21–9.02). The overall tertiary structures, which were defined by four endonexin domains containing calcium binding sites, were well preserved (Fig C in S1 File, Table C in S2 File). However, the primary amino acid sequences diverged significantly, with the lowest amino acid identity of 20.9% between STANN4 and STANN5. Groups with higher similarities were identified, such as STANN3.1, STANN3.2, and STANN3.3. Annexins 3.2 and 3.3 were the most closely related with amino acid identities of 90.5% and 70.1% with STANN3.1, respectively. STANN3.2 and 3.3 differed in length (302 and 317 aa, respectively) due to lack of the 14-3-3 like domain on the C-terminus of STANN3.2. Similarly, the N-terminal end of STANN3.2 and STANN3.3, but not STANN3.1, contained a putative myristoylation motif (MG). To date, a myristoylation-mediating membrane localization has been confirmed only for mammalian AnxA13b. With respect to plant annexins, a myristoylation motif was found in poplar annexin EEE95606.1, but the functionality of this motif was not experimentally verified. In summary, despite extensive similarities, there were substantial differences between members of the STANN3 subfamily. This suggested that family might be unique to *Solanaceae*, and that distinct cellular functions evolved for each of the annexins.

The potato annexins contained canonical type II calcium binding sites G-X-GTD-{30–40}D/E solely in the first and occasionally fourth endonexin domains (Fig C in S1 File). STANN4 and STANN8 appear to have lost calcium responsiveness as a result of substantial mutations (substitutions and insertions) in these regions. The calcium binding site in the fourth endonexin repeat was probably the only one preserved in STANN5. Tryptophan residues within the first endonexin repeat (G-W-GT) were conserved in potato annexins 1, 2, 8, and 9, but were replaced with phenylalanine (STANN5) or lysine (STANN3.1–3.3 and STANN4) in other annexins (Fig C in S1 File). This phenylalanine modification is not predicted to interfere with calcium binding because phenylalanine and tryptophan residues are both hydrophobic and possess aromatic rings. By contrast, the lysine modification may impede membrane translocation of annexin because introducing a positive charge into the calcium coordination site has the potential to disrupt calcium binding. Other amino acids or motifs important for the plant annexin tertiary structure were preserved in the potato proteins, such as histidine 40 (except in STANN3.2 and STANN4), cysteine 111 (except in STANN4), and cysteine 239 (except in STANN1).

### Potato annexin gene expression during drought

**Fig 2:**
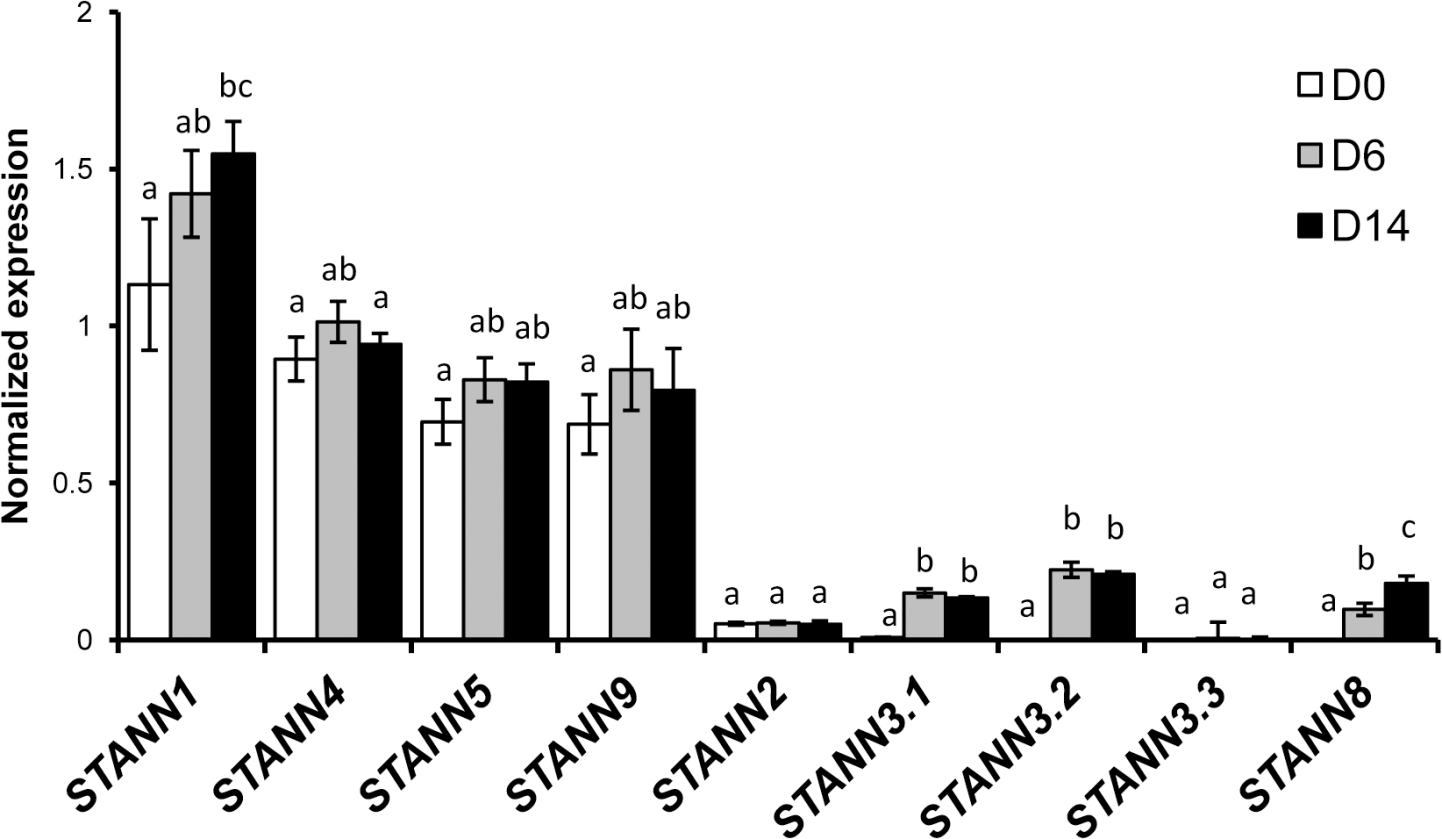
Profiling of annexin expression in WT potato leaves during drought. Potato WT plants grew in the walk-in growth chamber under controlled conditions. After 8–10 weeks irrigation was gradually reduced to decrease the field capacity (FC) to 25% (which took approximately 10 days) and then maintained at this level till 14^th^ day. Samples were collected from the first fully developed composite leaf from the top at indicated time points (D0 – beginning of drought, D6 – sixth day of drought, and D14 – fourteenth day of drought). RNA was isolated with Trizol and sq-RT-PCR was performed with primer sets specific for certain annexins. The level of expression was normalized against *EF1a* mRNA. Results are means ±SE (n≤4). Homogenic groups are determined by Tukey HSD (Honestly Significant Differences) test. The same letters designate values belong to the same homogenic group (p<0.05). Experiment was repeated twice.

To generate drought-tolerant potato, the genes whose products confer drought tolerance have to be identified. Potato annexins are a multigene family; therefore, we characterized the expression of all annexins during drought. Only five annexin genes (*STANN1*, *STANN4*, *STANN5*, *STANN9*, and *STANN2*) were expressed in the leaves of well-watered control WT plants (Fig 2). At the onset of drought (D0), *STANN1* mRNA was the most abundant transcript (relative to the *EF1a* mRNA). Over time, the level of *STANN1* mRNA increased whereas *STANN4*, *STANN5*, *STANN9*, and *STANN2* mRNA levels remained unchanged. The difference in the accumulation of *STANN1* mRNA from D0 to D14 was statistically significant (Fig 2). Concurrently, additional annexins were expressed that were not detected under control conditions. The levels of *STANN3*.*1* and *STANN3*.*2* mRNA (relative to *EF1a*) increased on D6 and remained elevated until the end of the drought period. The level of *STANN8* mRNA increased continuously during the whole period of water deficit (Fig 2). However, these induced annexins were expressed at levels at least ten-fold lower than that of annexin 1. This strongly suggested that STANN1 was the key annexin involved in the plant cell response to drought.

### Tolerance to soil water deficit

**Fig 3:**
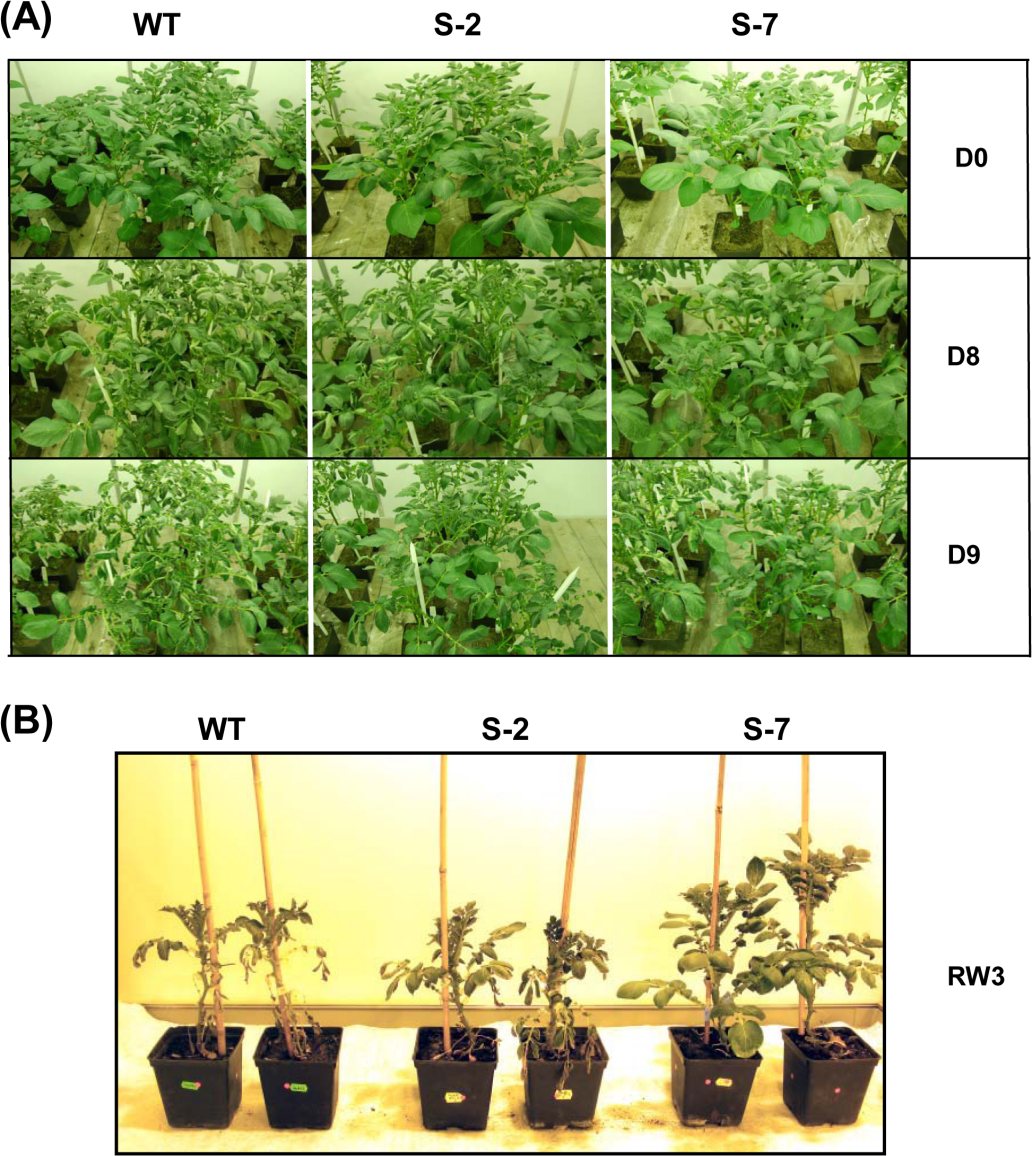
Drought tolerant phenotype of transgenic plants. Potato WT plants and transgenic lines (S-2, S-7) was subjected to drought as described above in Fig 2. (A) Drought stress phenotype of WT (left column), S-2 (middle column) and S-7 (right column) plants. Photographs were taken on the beginning (D0), on eighth (D-8) and ninth (D-9) day of drought. Experiments were repeated twice in greenhouse and twice in growth chamber and gave similar results. (B) Regeneration of potato plants after prolonged drought. The procedure of drought imposition was the same as described above but the FC was maintained at 25% until the twenty first day of drought (D21). On D22 plants were rewatered and after draining of gravitationally bound water FC was kept up at 65%. Photograph was taken on the third day after rewatering. Left side—two WT plants; middle–two S-2 plants, and right–two S-7 plants. Experiments were repeated four times and similar results were obtained both in greenhouse and in growth chamber.

Drought is one of the most devastating environmental stresses in modern agriculture; it reduces global crop yields in developed and developing countries [56]. Continued efforts are required to obtain new crop varieties that assure food security. Annexins were shown to be a promising target in model plants; thus we wanted to verify if they can be used to improve stress tolerance in crop plants. To investigate the effect of STANN1 on drought tolerance in potato transgenic plants overexpressing STANN1 were generated by introducing the STANN1coding sequence under control of the 35S promoter. Transgenic plants displayed normal morphology without any discernible abnormalities and/or growth aberrations under well-watered conditions in growth chamber and in the greenhouse. Leaf turgidity was similar between transgenic and WT plants, which indicated that the leaf water status of WT and transgenic lines was comparable (Fig 3A, upper panel and Fig D in S1 File). During soil water deficit, STANN1 overexpression conferred sustained turgor maintenance, whereas leaf wilting was clearly visible on D8 in WT plants (Fig 3A, middle panel). In WT the effect of drought was more apparent by D9, and leaves began to shrivel, roll, and curl up. Younger leaves near the top the of the plant were most severely affected (Fig 3A, lower panel). Leaves of the transgenic lines S-2 and S-7 maintained turgor and did not show signs of dehydration. Rewatering restored leaf turgor and normal growth resumed within 1 day for transgenic plants and 3 days for WT (Fig 3B). After 2 weeks of drought, S-2 and S-7 leaves were less damaged then those of WT. Experiments were repeated four times in succeeding years, under greenhouse and growth chamber conditions, and in all cases similar results were obtained (Fig D in S1 File). The exact number of irreversibly damaged leaves varied between experiments depending on the drought severity (intensity and length). Damage was consistently significantly lower in transgenic lines than in WT. For example, survival rates after a 3-week drought were 12% and 82% for WT and S-7, respectively.

**Fig 4:**
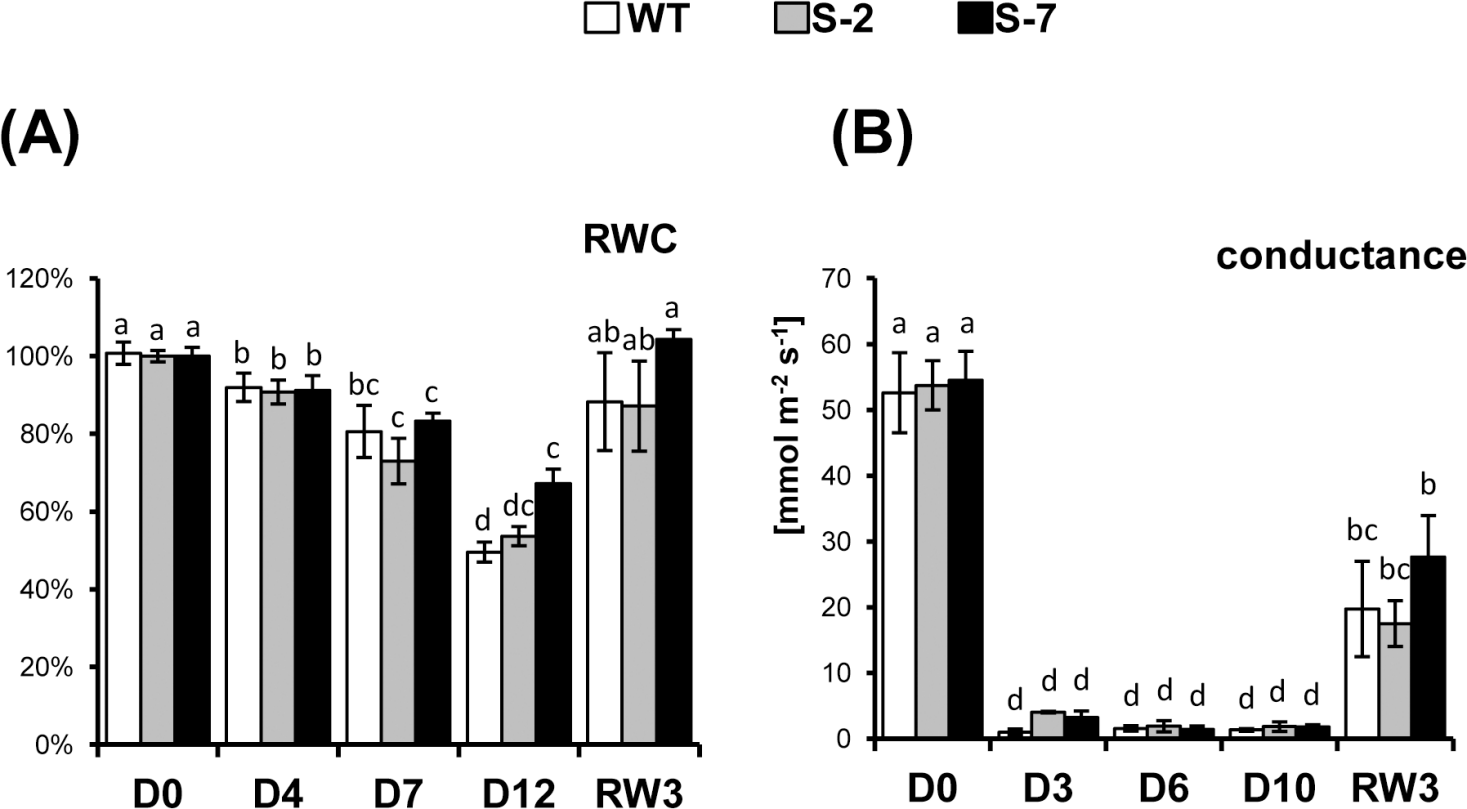
Examination of leaf water status. Potato WT plants (white bars) and transgenic lines: S-2 (gray bars) and S-7 (black bars) were subjected to 14-day drought as described in Fig 2. (A) Relative water content (RWC) analysis. Samples from the first fully developed undamaged leaf from the top of plant were collected at D0, D4, D7, D12 and 3 days after rewatering (RW3) and relative water content (RWC) was determined. Results are means ±SE (n = 3). (B) Stomatal conductance were measured in fully expanded, attached leaves at D0, D3, D6, D10 and RW3. After D10 the leaf surface was wrinkled to such an extent that further analysis was impossible. Measurements were done with a CI-510CF Chl fluorescence module, actinic light was provided by a CI-310LA light attachment. Results are means ±SE (n = 10). Experiment was performed three times and gave comparable results. Homogenic groups are determined by Tukey HSD (Honestly Significant Differences) test. The same letters designate values belong to the same homogenic group (p<0.05). Experiment was repeated 3 times and gave comparable results.

The ability to preserve turgor in leaves is closely related to drought tolerance. To further characterize drought responses in transgenic plants overexpressing STANN1, RWC changes under water deficit were analyzed. RWC was comparable in WT and transgenic plants. Drought reduced RWC in WT and transgenic plants. However, differences between lines became apparent with increasing drought severity and this became statistically significant at D12 (Fig 4A). Rewatering after 2 weeks of drought treatment restored control RWC values in WT and transgenic plants. The effect of drought on stomatal conductance (a measure of water and carbon dioxide vapor through the leaf stomata) was apparent by the third day after reducing watering, but the difference between WT and transgenic lines was statistically insignificant (Fig 4B). Conductance remained low during the whole period of water deficit and only partially returned to control levels on the third day after the resumption of watering.

The real goal for any genetic engineering efforts in crop plants is to improve crop yield. We examined the productivity of transgenic plants under control conditions and under drought. STANN1 overexpression improved plant yield both in terms of the total tuber mass and consistency of the tuber size (Fig E in S1 File). The net productivity of well-irrigated WT and transgenic S-7 and S-2 lines was almost identical but tuber quality (size and uniformity) was enhanced in the transgenic lines. A 14-day drought decreased the tuber yield of the WT plants by half, whereas yield loss for S-2 and S-7 lines under comparable conditions was statistically less significant. Tuber quality in the transgenic lines was less impaired after drought compared with WT. On the basis of these results, we concluded that increasing STANN1 levels is a promising strategy to improve drought tolerance in potato.

### Plant photosynthetic activity during drought

**Fig 5:**
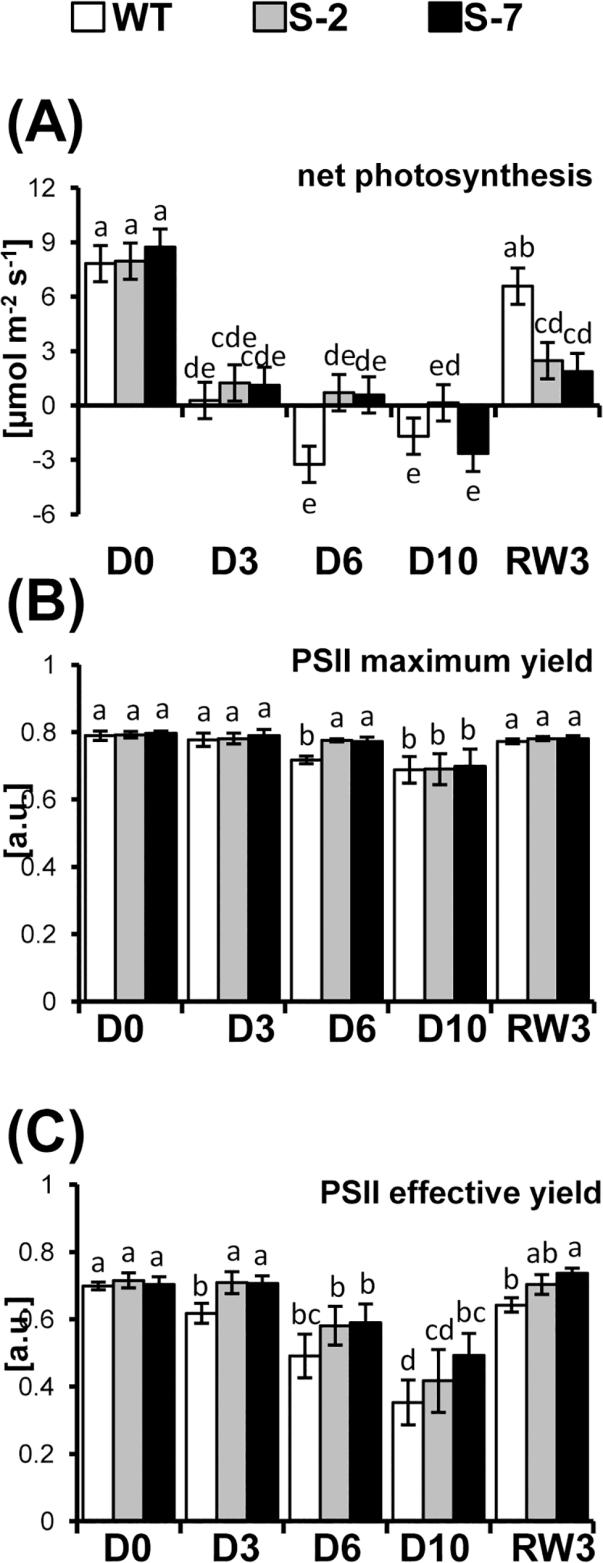
Netto photosynthesis and photosynthetic performance of PSII in potato plants during drought. Potato WT (white bars) and transgenic lines: S-2 (grey bars) and S-7 (black bars) were subjected to drought as described in Fig 2. (A) Netto photosynthesis, (B) maximum quantum yield of photosystem II (Fv/Fm) and (C) effective quantum yield of photosystem II,Y(II) were measured in fully expanded, attached leaves at D0, D3, D6, D10 and RW3. After D10 the leaf surface was wrinkled to such an extent that further analysis was impossible. Measurements were done with a CI-510CF Chl fluorescence module, actinic light was provided by a CI-310LA light attachment. Results are means ±SE (n = 10). Experiment was performed three times and gave comparable results. Homogenic groups are determined by Tukey HSD (Honestly Significant Differences) test. The same letters designate values which are not significantly different at p<0.05 and belong to the same homogenic group.

We showed that elevation of ATANN1 levels enhanced drought tolerance in potato, but the mechanism of this process was unknown. There is some indication that this could be due to annexin-mediated modulation of redox homeostasis. During drought, ROS accumulation in chloroplasts leads to oxidative damage of photosystems [57, 58]. PSII catalyzes water oxidation and provides electrons for all further photosynthetic reactions; thus, its efficiency is crucial for the entire pathway. Drought impairs photosynthetic capacity and reduces leaf netto carbon uptake due to increased photorespiration activity (another sink for the absorbed energy) [59]. To directly estimate the effect of STANN1 on drought-induced PSII damage, the photosynthetic performance of PSII in transgenic plants overexpressing STANN1 was characterized under drought conditions. Several physiological parameters related to plant vigor were analyzed to assess the effect of STANN1 overexpression. These included, net photosynthesis (Pnet, associated with plant vitality and biomass production) (Fig 5A), maximum efficiency of PSII in the dark-adapted state (a measure of the organization and vitality of PSII) (Fig 5B), and effective quantum yield of PSII in illuminated samples (Fig 5C). Under control conditions, STANN1 overexpression did not influence any of these parameters. By contrast, essentially all photosynthetic functions were disturbed during drought, and changes in the two overexpression lines were consistent.

WT Pnet declined to zero by D3 (Fig 5A). Subsequently, it dropped to negative values by D3 and D10. In the two transgenic plant lines, Pnet remained positive until D10. After rewatering, Pnet increased in all three lines (Fig 5A). Under control conditions, Fv/Fm values (Fig 5B) were similar in the all three plant lines (~0.79), and was in the same range as in most investigated plant species. Drought negatively affected Fv/Fm in our experiments; this was observed by D3 in WT, but become apparent in transgenic plants arent on D6. In all three lines, Fv/Fm recovered to baseline within 3 days of rewatering. Measurements were performed on upper non-wrinkled leaves, indicating that apical shoot meristems were not irreversibly damaged by dehydration. In WT and transgenic plants Y(II) (Fig 5C) declined steadily from the onset of drought, but the reduction appeared on D6 in transgenic plants, and the effect was significantly reduced compared to WT. Y(II) fully recovered in S-2 and S-7; however, even on the third day after soil resaturation, the physiological efficiency of PSII was not restored in WT. This suggested that photorespiration was activated later in STANN1 overexpressing plants then in WT. Thus, PETC was protected for a longer time against irreversible damage and diminished photorespiration-induced H_2_O_2_ accumulation in cytosol. These results show that PSII impairment in transgenic plants was fully reversible

### Photosynthetic pigments content in transgenic plants

Drought activates premature senescence in plants [60] and stimulates catabolism of photosynthetic pigments [61], particularly Chl and Car. We determined the photosynthetic pigment contents under drought conditions to better understand the effect of STANN1 on photosynthetic machinery.

#### Chl*a* and Chl*b* accumulation

**Fig 6:**
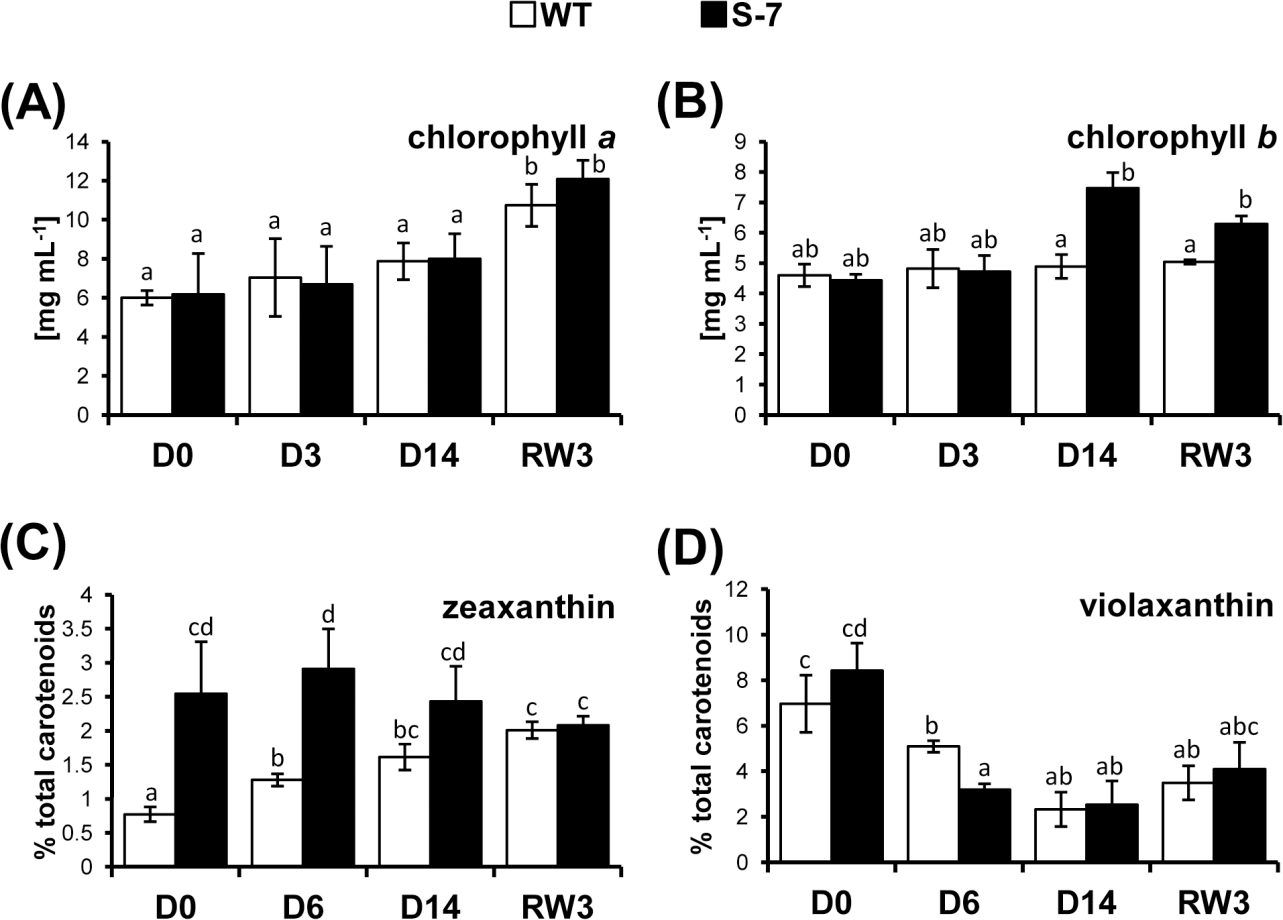
Photosynthetic pigment content during drought. WT (white bars) and transgenic line S-7 (black bars) were exposed to drought as described in Fig 2. Samples were collected at the same time during the day at D0, D6, D14 and RW3 from third, fourth and fifth fully expanded leaves from top at 4 hours after turning the light. The level (A) chlorophyll *a*; (B) chlorophyll *b*; (C) zeaxanthin; and (D) violaxanthin were determined Non-polar lipids were separated on an ACQUITY UPLC system (Waters) and peaks were integrated at 436 nm. The level of xanthophylls is expressed as percent of the total carotenoids. The level of chlorophyll is expressed as mg mL^-1^. Results are means ±SE (n = 3). Homogenic groups are determined by Tukey HSD (Honestly Significant Differences) test. The same letters designate values belong to the same homogenic group (p<0.05).

In WT and transgenic lines under well-watered conditions, the total Chl content (11.2±0.01 and 10.6±2.29 mg mL^-1^, respectively) and the ratio of Chl*a* to Chl*b* were similar. In WT accumulation of Chl*a* (Fig 6A) and Chl*b* (Fig 6B) did not change during drought and after rewatering, the level of Chl*a* increased to 180% of the control value at D0. During water deficit in S-7 line, the Chl*a* level was stable; however, Chl*b* levels increased and reached 168% at D14 compared to D0. Consequently, the Chl*a*/*b* ratio rose to 2.0. After rewatering, Chl*a* levels doubled and Chl*b* levels remained stable.

#### Xanthophyll (XCar)accumulation

The relative XCarabundance in the total Car pool changes during the day depending on the incident light [62]. To exclude diurnal fluctuations, samples were collected at the same time (approximately 4 h after the start of daily illumination). Under non-stress conditions, STANN1 overexpression did not significantly affect the total Car level, but the XCar content increased-zeaxanthin (Zea), 188%; violaxanthin (Viol), 144%—compared with that in WT plants (Fig 6C and 6D). This result indicates that the XCar cycle activity was higher in transgenic plants than in WT plants under the same light conditions. In WT plants, Zea content increased progressively during drought and reached a similar level to that in transgenic S-7 plants only after rewatering {0.35±0.01 pmol/g fresh weight (FW)}. In transgenic S-7 plants, the Zea level remained largely stable and fluctuated in the range of 0.31–0.34 pmol/g FW. Viol declined significantly during drought in both plant lines. The most significant reduction was observed during the first 6 days of drought, and was more pronounced in S-7 than in WT (57% and 10.5% reduction, respectively). At subsequent time points, the differences between lines disappeared and Viol remained at a stable level after rewatering (0.45±0.01 pmol/g FW in WT and 0.44±0.06 g^-1^ FW in S-7).

### NPQ activity

**Fig 7:**
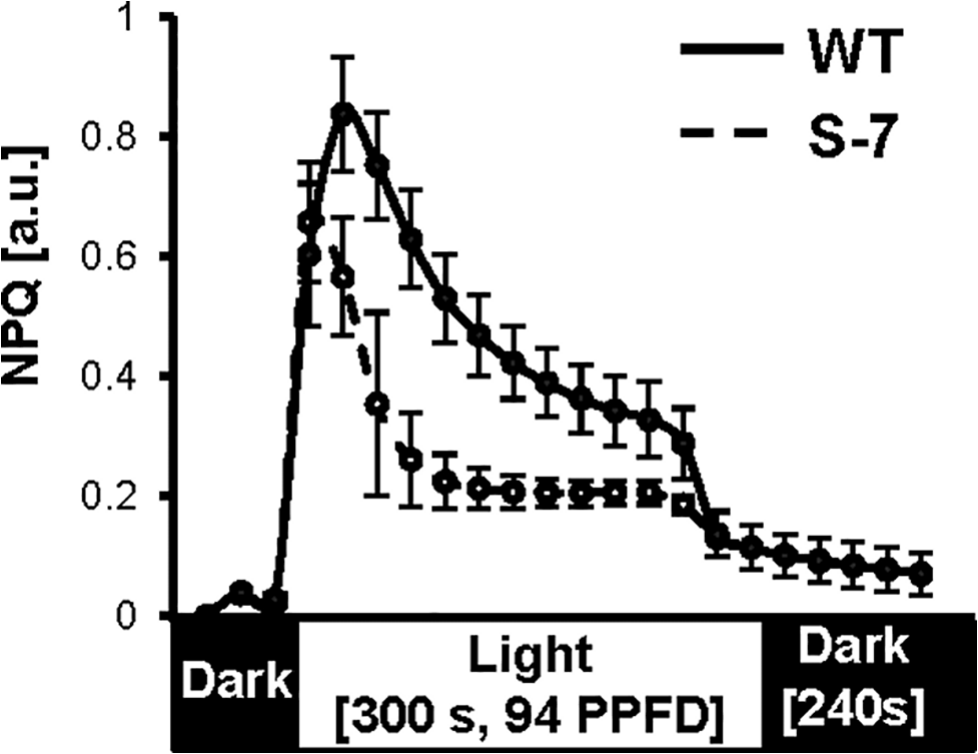
NPQ assayed in leaf of well-watered potato plants. Potato WT (dashed line) and transgenic S-7 (solid line) grew in the walk-in growth chamber under controlled conditions and were watered to maintained FC at 65%. Performance of gross non-photochemical quenching (NPQ) were assayed on the first fully developed composite leaf from the top of plant at 4 hours after turning the light with Dual PAM-100. For measurement plants were adapted to dark for 20 minutes and then stimulated with repeated light pulses of actinic light (94 PPFD) for 5 minutes and once again subjected to dark for 6 minutes. Each point represents the mean ±SD (*n* = 3–4). Experiment was repeated three times and gave comparable results.

The PSII complex is a highly vulnerable structure that undergoes constant photodamage even under optimal conditions and mediates plant adaptation to the dynamic light environment. NPQ is an effective short-term mechanism that provides protection for PSII against excessive irradiation and allows excess excitation energy to be harmlessly dissipated as heat [63]. For efficient NPQ, XCar cycle effectiveness and the level of PsbS protein are essential. The observed differences in XCar accumulation prompted us to analyze gross NPQ performance in attached leaves of control, well-watered WT, and transgenic plants (Fig 7). As expected, NPQ of S-7 and WT differed. Maximal NPQ occurred in S-7 plants after the start of daily illumination, and NPQ amplitude was ~25% higher in S-7 than in WT. The steady-state NPQ level was elevated and saturation was delayed in S-7 compared with those of WT. The chlorophyll a/b- binding photosystem II 22kD subunit S (*PSBS*, another key NPQ factor) mRNA level during drought was higher in S-7 than in WT plants (Fig F in S1 File). These results indicate that the NPQ capacity in transgenic line S-7 was greater than that of WT, which likely conferred better protection of PSII against photooxidative damage. While excess absorbed electrons were redirected to H_2_O_2_ in WT plants, they were more efficiently dissipated as heat in transgenic plants, which prevented subsequent ROS accumulation.

### Annexin overexpression affects hormonal homeostasis in plants subjected to drought

**Fig 8:**
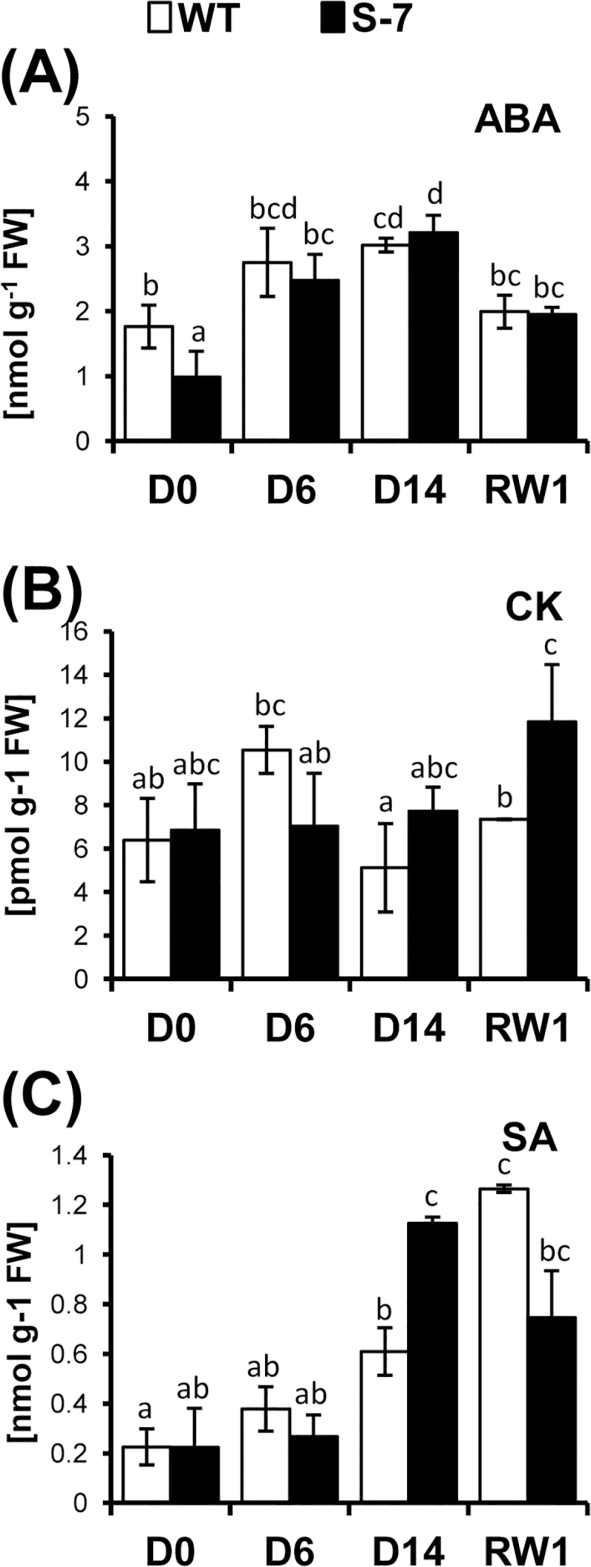
Accumulation of stress-related hormones during drought. WT (white bars) and transgenic line S-7 (black bars) were subjected to 14-day drought as described in Fig 2. The level of (A) absicisic acid ABA; (B) sum of active cytokinins, CK; (C) salicylic acid, SA were determined at D0, D6, D14 and RW1. Samples (0.5g of fresh leaf tissue without the midrib) were collected from the first fully developed, undamaged leaf from the top of plant at 4 hours after turning the light. Labeled internal standards were added to the leaf samples before homogenization. Hormones were then extracted, purified using a SPE-C18 column and separated on a reverse phase-cation exchange SPE column. Hormones were quantified using a hybrid triple quadruple/linear ion trap mass spectrometer. The level of ABA and SA is expressed as nmol g^-1^ of fresh weight; the levels of cytokinins–as pmol g^-1^ of fresh weight. Results are means ±SE (n = 3). Homogenic groups are determined by Tukey HSD (Honestly Significant Differences) test. The same letters designate values belong to the same homogenic group (p<0.05).

The drought phenotype of transgenic potato plants overexpressing STANN1resembled that of plants overproducing cytokinins (CK). Compelling evidence indicates that the redox signaling network integrates with phytohormone-activated pathways [64]. ROS are positioned upstream and downstream of at least some hormone-signaling pathways [65]. We therefore stress-hormone levels {pro-senescing: ABA and salicylic acid (SA); anti-senescing: CK} in leaves of WT and S-7 plants subjected to drought. Under well-watered conditions, the level of biologically active ABA in transgenic plants was significantly lower than in WT (Fig 8A). However, this difference was insignificant by D6 after the initiation of drought This suggested that biosynthesis of ABA in transgenic plants during the first week of drought was more active than that in WT, which is consistent with a more pronounced reduction of Viol (ABA precursor) levels in transgenic plants (Fig 6D). During the second week of water deficit, only a slight increase in ABA level was observed and maximum levels on D14 were similar WT and S-7 (3.21±264.01 and 3.02±101.59 nmol g^-1^ FW, respectively) (Fig 8A). As expected, ABA levels declined to control values on resumption of watering.

Under control conditions, annexin overexpression had no significant effect on CK levels (Fig 8B and Table D in S2 File). The contents of active and total CK were similar and amounted to 6.35 and 6.90 pmol g^-1^ FW, and 506.34 and 542.08 pmol g^-1^ FW, in WT and S-7 plants, respectively. Drought stress was associated with down-regulation of *trans*-zeatin (tZ), the most physiologically active CK involved in the stimulation of cell division. During early drought stages (RWC *~*85%, only minor difference from control conditions), the level of active CK in WT increased, especially compared to the less active isopentenyladenosine (iPR) levels. Active CK declined under severe drought conditions, with the exception of *cis*-zeatin (cZ) and its riboside (cZR), both of which were CK species associated with stress responses. After rewatering, active CK content strongly increased, especially that of *trans*-zeatin (tZ), whereas cZ and cZR levels substantially declined. High levels of active CK (including high levels of cZ) were maintained in S-7 even under severe drought conditions. These levels were substantially higher than in parental plants. After rewatering, active CK elevation was much more pronounced in S-7 than in WT. The level of storage compounds (CK O-glucosides) was generally low. By contrast, levels of deactivation products (CK N-glucosides) substantially increased during drought, probably as a result of the enhanced deactivation of CK (data not shown).

SA accumulation was reported in response to different abiotic stresses [66]. STANN1 overexpression had no effect on SA levels under well-watered conditions. SA accumulation in WT and S-7 did not change significantly under moderate drought (D6). During the second week of water limitation, the SA level increased in both lines, and SA accumulation in S-7 was approximately twice that in WT (Fig 8C). The SA level declined in S-7 during recovery, but remained slightly higher than that observed at D0. By contrast, SA continued to increase in WT and exceeded the level observed in S-7. These data indicate that ROS-modulating systems are activated more rapidly and to a higher extent in transgenic plants overexpressing STANN1 than in WT plants.

In summary, genetic modification influence neither ABA synthesis no ABA-dependent responses. The elevation in CK metabolism upon rewatering was consistent with phenotypic observations. SA levels in S-7 increased rapidly during drought and peaked by D14 but declined rapidly after rewatering. This suggested that SA-mediated activation of antioxidant systems during drought was faster in STANN1 overexpressing plants. In WT plants, delayed SA-mediated effects such as induction of PCD might be induced.

### STANN1 mitigates drought-mediated oxidative stress in cytosol and chloroplasts

Although the experimental plants were grown under constant temperature conditions, heat stress response (HSR) was induced in WT and transgenic plants during drought. In WT plants, water deficit increased the accumulation of chloroplast-specific *HSP100* and cytosol-targeted *HSP40* mRNAs (compared to the EF1a normalization control), which peaked during the second week of drought. In transgenic plants, only *HSP100* expression was induced under water deficit (Fig F in S1 File). This result suggests that STANN1 overexpression mitigates cytosolic oxidative stress.

### STANN1 mitigates photooxidative stress induced by MeV

Enhanced stress tolerance frequently reduces plant responsiveness to light [67]. The chloroplast antioxidant system is “loosely tailored” to maintain an endogenous ROS pool under control conditions [12], which enables plants to quickly respond to fluctuating light levels. Consequently, significantly improving ROS scavenging enhances protection against sustained stress, but also desensitizes plant light responses and impairs environmental fitness. To verify if annexin-mediated drought tolerance influenced light responses, we analyzed the effect of the photosensitizer MeV on transgenic plants overexpressing STANN1. MeV induces oxidative stress, which enables studies of oxidative tolerance and stress cross-tolerance in plants [68]. MeV induces an oxidative burst by accepting electrons from PSI and transferring them to molecular oxygen, which results in massive H_2_O_2_ accumulation in light and generates oxidative stress in chloroplasts.

#### Leaf disc senescence assay

Leaf discs from WT, S-2 and S-7 plants were exposed to normal light (150 PPFD) in the presence of 10 and 50 μM MeV. The damage caused by MeV was visualized as the degree of leaf tissue bleaching. In the absence of MeV, exposure to light for up to 30 h had no significant effect on leaf discs. By contrast, exposure to light during MeV treatment induced leaf tissue bleaching, which increased according to MeV concentration (Fig G in S1 File). Transgenic plants S-2 and S-7 had higher tolerance to MeV, and exhibited lower levels of leaf disc bleaching in light.

#### Quantification of ROS and lipid peroxidation

**Fig 9:**
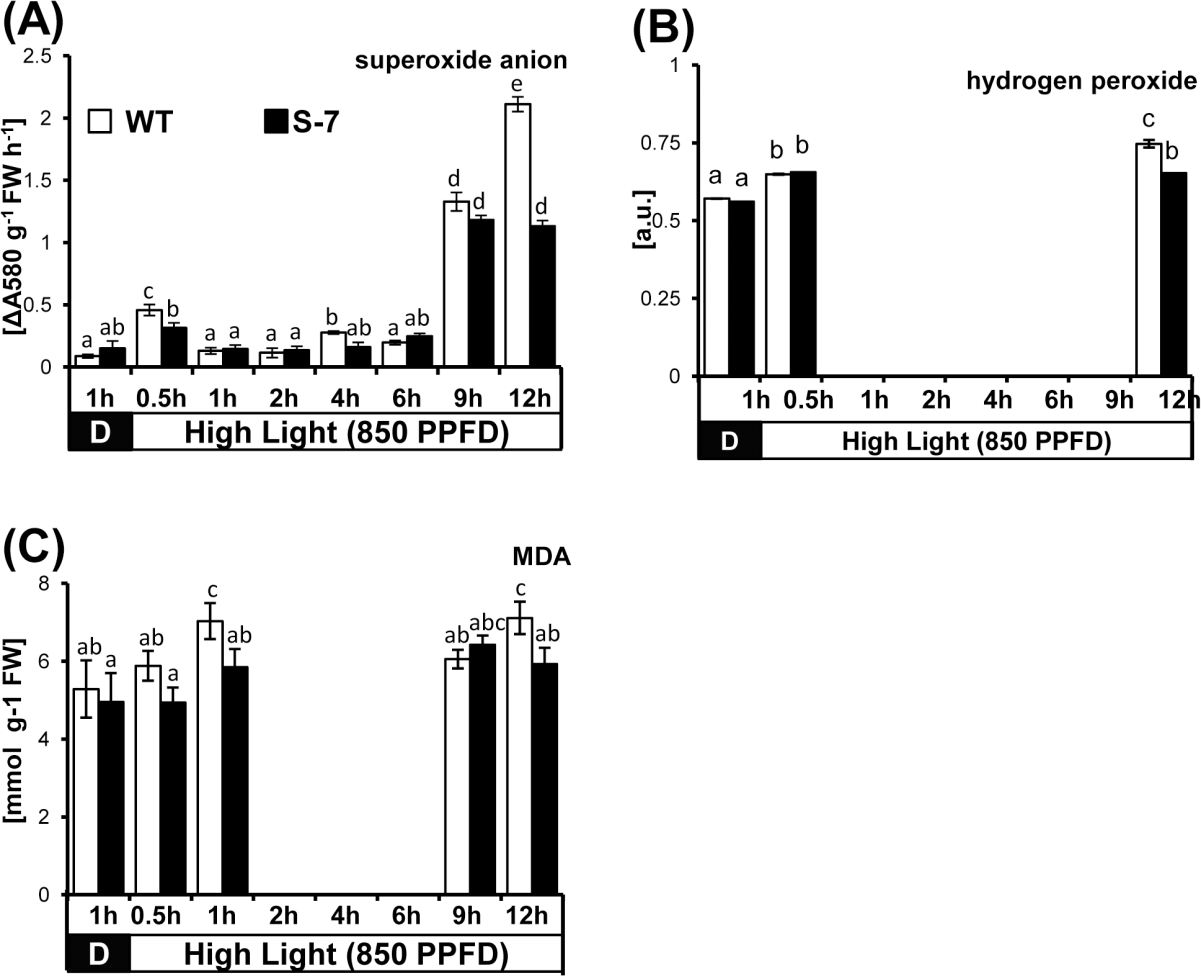
Accumulation of ROS (hydrogen peroxide and superoxide anion) and lipid peroxidation. Potato WT (white bars) and transgenic line S-7 (black bars) grew in walk-in growth chamber under controlled conditions. Leaf discs were expunged from the third, fourth and fifth upper fully expanded leaves and immediately vacuum infiltrated with methyl viologen (50 μM). After 1 hour incubation in dark discs were exposed to high light irradiance (850 PPFD) for indicated times (0.5–24 hours). Superoxide anion was determined colorimetrically with nitro blue tetrazolium chloride 9NBT). Hydrogen peroxide was stained in tissue with diaminobenzidine tetrahydrochloride (DAB) and quantified using the ImageJ. Lipid peroxidation was estimated spectrophotometrically with thiobarbituric acid (TBA). Results are means ±SE (n = 5). Homogenic groups are determined by Tukey HSD (Honestly Significant Differences) test. The same letters designate values belong to the same homogenic group (p<0.05). Experiment was repeated twice.

To further analyze STANN1-mediated protection against light stress, leaf discs from WT and S-7 plants were subjected to the combination of relative excess light (850 PPDF) and 50 μM MeV. The levels of superoxide anion, hydrogen peroxide, and malonyldialdehyde (MDA) were quantified at the indicated time points (Fig 9).

Exposure of WT to excess light and high MeV concentration induced biphasic accumulation of superoxide anions, with an initial peak at 30 min after induction and a second, more substantial and long-lasting, peak beginning at 9 h after induction. In S-7, an initial increase in superoxide anion level was observed, which was significantly lower than that in WT. The maximum level of O_2_^-^ was the same in WT and S-7, but the kinetics of the second peak differed (Fig 9A). In WT, the level of superoxide increased steadily from 6 to12 h after induction. In S-7 superoxide anion accumulation occurred during 6–9 h after induction, reaching a similar maximal level as in WT at this time point, and the superoxide level then remained unchanged until 12 h after induction.

In WT, light-induced changes in H_2_O_2_ level were biphasic, with a second higher and sustained peak (Fig 9B). The first peak occurred within 30 min and the second peak occurred by 12 h after induction. In S-7, the first peak had a similar magnitude to that in WT. After several hours, no further accumulation of H_2_O_2_ was observed in S-7, and overall levels were significantly lower than in WT.

Lipid peroxidation, measured as an MDA equivalent, was apparent in WT only after 30 min and 12 h. No statistically significant changes in the lipid peroxidation state were observed under high light stress in S-7 (Fig 9C).

### Annexin 1 attenuates cell death and protects chloroplast structure against oxidative stress

**Fig 10:**
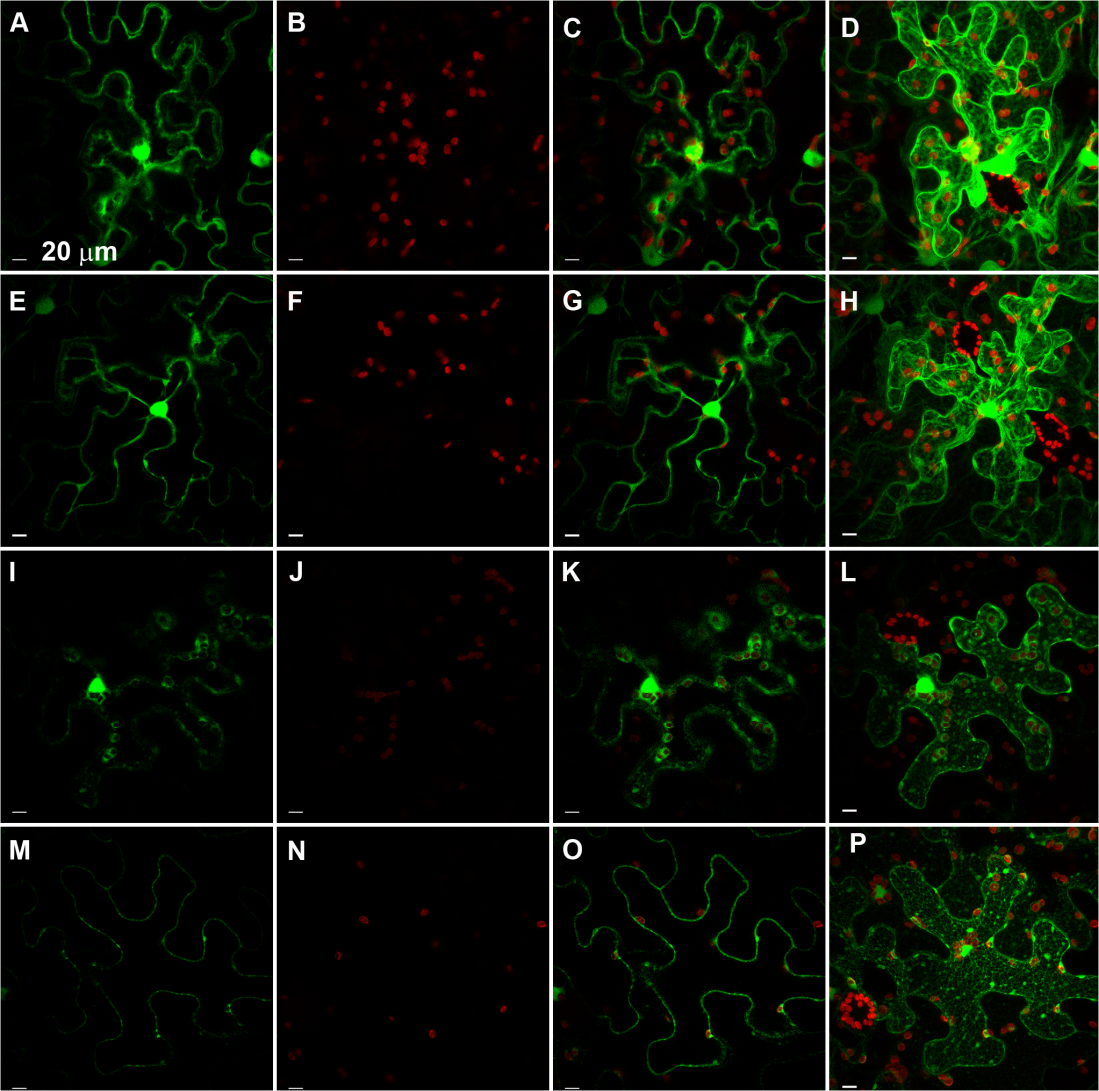
STANN1 attenuated MeV-induced photooxidative stress. Confocal laser scanning image of the leaf epidermis of tobacco plant transiently expressing GFP (A-D and I-L) or STANN1_GFP (E-H and M-P). 3 days after infiltration leaf discs were excised and subjected to high light (850 PPFD) (A-H) or the combine treatment of high light (850 PPFD) and 50 μM MeV (G-L). The fluorescence was monitored with Nikon TE-2000E EZ-C1 exc. 488 nm and emission 515/30 and 605/75 for GFP and chloroplast, respectively. First column represent single focal plane, second–chloroplast autofluorescence acquired with the same excitation parameter for each construction to visualized the difference between responses to the same treatment, third–overlay of green and red fluorescence channels with GFP enhanced to visualized cells; right column–stack obtained with Volume Render program EZ-C1 combined with chloroplasts. Scale bar is 20 μm. Experiment was performed 3 times.

In our experiments, the annexin STANN1 attenuated both phases of chloroplast-derived oxidative stress. In transgenic plants overexpressing STANN1, the expression of nucleus-encoded PSII proteins (Fig F in S1 File) and HSPs was modified correspondingly (Fig F in S1 File). A transient mGFP expression assay was performed to confirm that tolerance to photooxidative stress was due to elevated STANN1 levels. In this experiment, STANN1 was produced as an in-frame C-terminal fusion with mGFP. *N*. *benthamiana* leaf discs were transformed with STANN1_mGFP (experiment) or mGFP (control) constructs. Leaf discs were then subjected to high light or to the combination of high light and MeV as described above. Leaves with similar fluorescent protein expression levels were used for analysis. Exposure to high light alone had no effect on cell structure, regardless of the construct used (mGFP-alone, Fig 10A and 10B and 10C and 10D; STANN1_mGFP—Fig 10E and 10F and 10G and 10H). High light plus MeV induced cytosol condensation and chloroplast damage (as determined by a decline in chloroplast autofluorescence) in mGFP-expressing cells (Fig 10I and 10J and 10K and 10L). Annexin 1 overexpression attenuated both of these effects, and the cell morphology resembled that of control samples (Fig 10M and 10N and 10O and 10P). Chloroplast fluorescence intensity was quantified and, there was no significant difference in mGFP fluorescence between plants transiently expressing mGFP and STANN1_mGFP. The difference in chloroplast autofluorescence (red) between the mGFP and STANN1_mGFP expressing leaves was statistically significant. This strongly suggests that the chloroplast structure was maintained in the presence of STANN1 protein.

## Discussion

This study clearly demonstrates that elevation of endogenous STANN1 expression can be successfully employed to improve potato tolerance to water deficit. Under optimal conditions, genetic modification had no negative effects on plant phenotype, growth, or productivity. Reduction of the photosynthetic rate in response to water deficit is usually attributed to ROS-induced damage of lipids, pigments, and proteins in the photosynthetic apparatus. Overexpressed STANN1 relieved the negative effects of drought stress, such as degradation of photosynthetic pigments, reduction of photosynthetic activity, and loss of productivity. In transgenic plants, NPQ was induced more rapidly and had higher capacity in STANN1-overexpressing plants, which contributed to increased tolerance to photooxidative stress. Exposure to MeV reduced ROS accumulation and membrane lipid damage, so STANN1-overexpressing plants were not desensitized to light. Consequently, we assume that maintenance of photosynthesis during water deficit was due to protection against drought-induced oxidative stress and/or modification of redox/hormonal signaling in STANN1-overexpressing plants. We propose that manipulation of annexin expression is a valuable new approach for crop improvement that focuses on delay and/or attenuation of leaf senescence and maintenance of physiological processes when plants are exposed to challenging environmental conditions.

### Annexin selection for transgenic experiment

Potato annexins have similar tertiary structures but display different levels of primary amino acid sequence similarity. Despite some extensive structural similarities, the individual annexins displayed unique expression patterns in the different plant organs (data not shown) and in response to drought. This suggests that the specialization of individual family members towards unique roles in growth/development or adaptation to environmental conditions. Indeed, recently it was shown that functional knock-out of annexin 5 (At1g68090) in Arabidopsis was male-sterile due to the abortion of pollen grains before mature pollen stage; however, on the basis of primary amino acid sequence, no specialized functions could be predicted for annexin 5. Detailed investigations revealed that ATANN5 is the most abundantly expressed annexin during microsporogenesis [69, 70]. It will be necessary to test if ectopic expression of any other Arabidopsis annexin under the ATANN5 promoter restores pollen development.

Expression of four potato annexins was induced during drought. However, STANN1 expression was several-fold higher than other annexins. This strongly suggested that STANN1 was involved primarily in stress responses and argued against complementation within the annexin family, at least at the transcriptional level. *STANN1* is expressed in all plant organs (data not shown); therefore, the risk of inducing developmental aberrations due to ectopic *STANN1* expression during development is minimal. Hence, we considered STANN1 as a good candidate to improve drought tolerance in potato and possibly other crops.

### STANN1 mitigates chloroplast-induced oxidative stress in cytosol

In light, chloroplasts are one of the major stress-induced ROS sources in plant cells [11]. Abiotic stresses reduce CO_2_ assimilation, which results in over-reduction of the PETC [71]. Under these conditions, oxygen can be utilized instead of NADP^+^ as an alternative acceptor for excess electrons [13]. Disruption of chloroplast redox poise permeates throughout the cell and activates secondary ROS sources in other compartments. In mesophyll cells of *Eupatorium adenophorum*, tenuazonic acid (TeA) inhibits electron flow along PSI and PSII and induces H_2_O_2_ accumulation in chloroplasts within 1 h. By 4 h, H_2_O_2_ spread to the cell walls facing intercellular spaces [72]. The most prominent secondary ROS source is the membrane NADPH oxidase complex. ABA induces RBOH gene expression in Arabidopsis leaves and guard cells [14, 73], the *Hordeum vulgar*e aleurone layer [74], and *Zea mays* seedlings [75]. Furthermore, NADPH oxidase-mediated ROS accumulation has been reported in ozone-treated Arabidopsis leaves [76, 77] and salt-treated Arabidopsis root tips [78].

We provided evidence that photooxidative stress in potato leaves induced a biphasic oxidative burst, with the first transient peak after 1 h and the second more significant peak occurring by 12 h. In STANN1-overexpressing plants, both phases of ROS accumulation were reduced. Biphasic ROS accumulation with a similar kinetics was reported in response to ozone and salt stress treatments [76–78]. In Arabidopsis and tobacco, the first transient ROS peak occurring after O_3_ treatment originated in the chloroplast, whereas the second required NADPH oxidase activity and undisturbed functioning of PETC [77].

The question arises as to how annexins that contain no specific chloroplasts signal sequences can modulate processes inside chloroplasts and attenuate the first peak. Annexins are found occasionally in chloroplast proteomes of some plants (reviewed in [16]). For example, a mustard (*Sinapis alba* L.) annexin was identified as a component of a multisubunit chloroplast RNA polymerase A complex [79]; however, these results were not confirmed in a subsequent study [80]. Overall, these chloroplast localizations remain exceptions. We believe that annexin-mediated protection of chloroplasts and photosynthesis could be an indirect effect of increased redox homeostasis buffering in the cytosol. We proposed that the first peak of ROS accumulation during photooxidative stress in potato is primarily due to chloroplast-generated ROS, whereas the second peak results from activation of secondary ROS sources, and STANN1 overexpression protects chloroplasts by improving ROS-scavenging systems in the cytosol.

In Arabidopsis, deletion of cytosolic ascorbate peroxidase 1 (APX1) resulted in collapse of chloroplast ROS-scavenging system; this induced degradation of thylakoid and stromal/mitochondrial APXs, a cytochrome b6f complex subunit protein, and the small subunit protein of Rubisco [81]. At the same time, chloroplast APX is one of the very first targets for ROS-mediated inhibition, and ROS accumulation rapidly reduced chloroplast antioxidant capacity [82, 83]. In Arabidopsis and tobacco plants, enhancing the antioxidant capacity of chloroplasts and cytosol has a beneficial effect on photosynthesis and stress tolerance [84], whereas removal of any single antioxidant component reduces photosynthesis and stress tolerance [81, 85–88]. Manipulation of chloroplast/cytosol antioxidant capacity was successfully used to modulate potato tolerance to adverse conditions. Transgenic potato lines engineered to express cytosolic or chloroplast Cu/Zn-superoxide dismutase (Cu/Zn-SOD) from tomato displayed enhanced tolerance to MeV [89]. Overexpression of cytosolic Cu/Zn-SOD from *Potentilla atrosanguinea* improved drought stress tolerance and enhanced net photosynthetic rates [90]. Co-expression of Cu/Zn-SOD and APX in chloroplasts enhanced potato tolerance to multiple abiotic stresses, including chilling, high temperature, photooxidative stress, and drought [91]. Accordingly, the lack of chloroplast thioredoxin CDSP32 resulted in greater susceptibility of potato plants to oxidative stress [92]. Taken together, these data show that elevating cytosolic antioxidant capacity is a promising way to enhance stress tolerance in potato. STANN1 overexpression improved drought tolerance and mitigated photooxidative stress, similarly to that observed for plants with overexpression of ROS-scavenging enzymes. The accumulation of mRNAs coding for cytosolic HSP40 and chloroplast HSP100 was entirely or partially reduced during drought, suggesting that the HSR in transgenic lines developed more slowly and to a lesser extent. This is in agreement with a previous report [46] for *Solanum andigeanum*, in which the expression level of respective *HSP* mRNAs during drought was higher in less tolerant lines than in more resistant landraces.

In summary, we assume that annexin-mediated protection of chloroplasts from ROS-induced damage is of utmost importance for plant stress resistance and recovery. Chloroplasts host crucial biosynthesis pathways (e.g., of hormones, Car, amino acids, and lipids) and are the site of cross-talk between basic metabolic pathways and stress responses, which places them in a key position with respect to coordination of defense responses [93, 94].

### Cross-talk between redox signaling and phytohormone-mediated pathways in transgenic plants overexpressing STANN1

Cross-talk between ABA, SA, CK, JA and other phytohormone pathways modulates plant development and stress adaptation. Our results showed that increased STANN1 expression modified drought-induced hormone accumulation. We assume that this is an indirect consequence of STANN1-mediated modulation of cellular redox homeostasis. Accumulating data indicate that multi-faceted and multi-level feedback interactions orchestrate hormone- and ROS-mediated signaling networks. Alterations in the cellular redox state were sufficient to modify hormone accumulation and their downstream effects [64]. ROS signaling is positioned upstream and downstream of hormone-signaling pathways [65, 95]. Redox cues integrate with the action of different phytohormones such as ABA and SA in the coordination of plant growth and stress tolerance [95, 96].

The control SA levels in transgenic plants were not significantly different than those in WT plants, and were similar to previously reported values for potato cv Desiree [97]. *S*. *tuberosum* has higher basal SA levels than Arabidopsis, maize, tobacco, or tomato [98]. Increases in SA levels in potato are relatively moderate (e.g., two-fold) after infection with *Phytophthora infestans*, compared with a 20-fold increase in Arabidopsis [99]. There is a lack of data on SA accumulation in potato leaves during drought, although it has been shown that SA functions as a regulatory signal mediating drought stress responses in several plant species [100, 101]. In our experiments, SA increases during water deficit in both plant lines were similar (6-fold in WT and 5-fold in S-7 over basal level), which was in perfect agreement with observations in *Phillyrea angustifolia* [102]. However, the SA peak was observed in WT plants only after rewatering, whereas in transgenic potato it occurred earlier, even during drought.

Recently, it became clear that SA is an important regulator of photosynthesis. In Arabidopsis, SA influences plant photosynthetic performance, and properly balanced SA levels are necessary for acclimation to changing light [103, 104]. The SA-mediated signaling pathway in Arabidopsis is involved in optimal photosynthetic activity under stress conditions by modulating redox homeostasis [105]. SA enhances the cell antioxidant capacity during drought, although the mechanism of this process is unclear. Endogenous SA deficiency in potato results in ineffective induction of stress defense system and enhances stress sensitivity [97, 106, 107]. During plant response to pathogen infection, SA inhibits the ROS-scavenging enzymes CAT and APX [108–110] and stabilizes H_2_O_2_ levels. SA and H_2_O_2_ function as a positive-feedback amplifying loop; if not properly balanced, this loop exerts detrimental effects for cell survival. In rice, reduced endogenous SA levels enhanced H_2_O_2_ accumulation and the appearance of spontaneous necrotic lesions during senescence and development of oxidative damage and in response to high light intensities [111]. SA accumulation induces different responses depending on the timing and accumulation level; it induces stress-responsive defense systems such as antioxidant enzymes, or induces PCD in response to long-term elevations of SA levels. In WT plants, slow and prolonged SA accumulation despite the resumption watering may ultimately lead to PCD. Rapid SA accumulation in transgenic plants appears to indicate more efficient mobilization of SA-induced stress responses, and accounts for improved photosynthetic performance.

Recent work shows that CK has an important role in plant adaptation to environmental stresses such as drought, cold, osmotic stress, and light stress [112–115]. In our experiments, CK species and their levels under control conditions were similar to those previously reported for potato cv Desiree [116]. STANN1 overexpression did not influence CK profiles or steady-state levels, but CK levels were maintained during water deficit and rapidly increased after rewatering.

CK antagonizes many ABA-induced physiological responses to drought such as stomatal closure or leaf senescence [117]. Maintaining CK biosynthesis during drought improves stress tolerance, confers protection against photooxidative stress, and mitigates reductions in photosynthesis [118–123]. CK activity is anti-senescent and associated with maintenance of greater antioxidant activity. In creeping bentgrass, elevated CK levels due to senescence-driven expression of isoprenyl transferase (IPT), a key enzyme in CK biosynthesis pathways, conferred drought resistance, increased the levels and activity of scavenging enzymes such as APX and CAT1, and reduced MDA accumulation [124]. Similarly, elevated CK levels in tobacco plant leaves and chloroplasts conferred higher physiological parameters than those in controls [125], and increased APX and dehydroascorbate reductase (DHAR) activity, which prevented over-oxidation of the chloroplastic ascorbate (ASC) pool. CK regulates stress responses on several levels, such as inducing stress-inducible gene expression [126, 127], including peroxidases, GRX, and glutathione S-transferases (GSTs). Plants with reduced CK levels had lower ROS-scavenging capacity, exhibited more severe photodamage after high light treatment, and had reduced neoxanthin and Zea levels under control conditions, which declined further during photooxidative stress [128]. Similar effects were observed in scavenging enzyme activities, and a strong reduction in APX and SOD activities were observed under control conditions and in response to light stress [128]. We assume that sustained biosynthesis of CK during drought in transgenic potato plants overexpressing STANN1 remediates oxidative stress and improves photosynthetic performance.

### STANN1 overexpression affects ABA accumulation and NPQ

ABA is a key factor in abiotic stress responses (induces stomatal closing and transcriptional reprograming); therefore, it is of utmost importance that ABA content and ABA-dependent stress signaling pathways in WT and transgenic plants are similarly activated. The ABA biosynthetic pathway is a side-branch of the Car biosynthetic pathway, with Viol being a direct precursor [129]. Viol is synthesized from Zea by zeaxanthin epoxidase, which is constitutively active in darkness and sub-saturating light; hence, under such conditions, the level of Viol far exceeds the level of its precursor. Instead, under saturating light (when the proton gradient produced is too high to be entirely consumed for CO_2_ assimilation), the level of Zea increases as a consequence of violaxanthin deepoxidase (ViolDE) activation, which requires reduced ASC as an electron donor. Hence, the actual level of Zea is determined mainly by the processivity of ViolDE [130]. Elevation of the annexin level resulted in reduced the ABA steady-state level and concomitantly increased the relative content of photoprotective Zea and Viol, which suggests that the ABA synthetic pathway was disabled. However, drought-induced ABA accumulation had similar kinetics, and similar maximal ABA levels were eventually achieved in transgenic and WT plants.

Both in control conditions and during drought, the levels of Viol and Zea were higher in STANN1-overexpressing plants than in WT plants. Partitioning of Viol into competing biosynthetic pathways (reconversion to Zea or ABA biosynthesis) depends on the chloroplast ASC status. The accessibility of reduced ASC promotes ViolDE activity resulting in Zea accumulation, whereas depletion of reduced ASC activates ABA biosynthesis. In leaves of ASC-deficient *vtc1* Arabidopsis plants, the ABA level is increased by 60% [131]. The Arabidopsis mutant *npq1* with no functional ViolDE does not accumulate Zea in HL. This is accompanied by increased photodamage of photosynthetic apparatus (reduction in CO_2_ assimilation and elevated lipid peroxidation) and strongly inhibited NPQ [132].

Overall, the plant capacity to dissipate excess light energy in a non-photochemical manner is also affected by redox poise. In leaves of two Arabidopsis ASC-deficient lines that over-accumulate ABA, *vtc1* and *vtc2-2*, NPQ at HL is decreased [133, 134]. Regeneration of the ASC pool is maintained mainly by DHAR [135] and suppression of DHAR in Arabidopsis results in lower induction of NPQ, while increased DHAR expression enlarges the size of the XCar pool [135]. For activity, DHAR requires glutathione as an electron donor, and in Arabidopsis *pad2-1* mutants, a shortage of glutathione also impairs NPQ and compromises adaptation to severe drought stress [136]. Similarly, an increased level of another lipid-soluble antioxidant, α-tocopherol, restores the control level of NPQ in the Arabidopsis *npq1* mutant lacking Zea [137]. Over-accumulation of α-tocopherol in the *Chlamydomonas reinhardtii* double mutant *npq1 lor1* (lacking both Zea and lutein) restores tolerance to HL and tolerance to oxidative stress [138]. These results suggest that NPQ is dependent on redox homeostasis, probably due to the effect on the xanthophyll cycle and Zea accumulation. Hence, an enhanced buffering capacity in the cytosol upon overexpression of STANN1 could result in improved NPQ.

### STANN1 affects redox homeostasis

Different hypotheses have been proposed to explain the molecular basis of annexin-mediated alleviation of oxidative stress, including innate peroxidase activity [21, 139, 140], calcium-induced stabilization of peroxidases activity [141], and modulation of calcium influx [142, 143]. Based on the results of our experiments, we assume that annexin-mediated reduction in oxidative stress in transgenic potato overexpressing STANN1 results from the annexin effect on thiol-disulfide homeostasis.

Downstream transmission of several environmental cues for H_2_O_2_ accumulation is sensed and mediated by several ROS-neutralizing systems, which are low-molecular-weight antioxidant buffers such as ASC and glutathione (GSH), and oxidoreductases such as GRX, TRX, and scavenging enzymes [144]. The most prominent ROS-scavenging and redox-signal perception system is GSH accumulation and GSH oxidation to disulfide (GSSG) during ASC regeneration in the glutathione-ASC cycle [145, 146]. This type of redox imbalance is transduced downstream by reversible formation of a mixed disulfide between GSH and a target protein (*S*-glutathionylation). An increased GSH:GSSG ratio was observed in plants exposed to chilling, heat stress, heavy metals, xenobiotics, drought, ozone, pathogen [147–153], and during oxidative stress resulting from deficiency in the H_2_O_2_-scavenging photorespiratory enzymes CAT or APX [88, 154–157]. In maize and rice, the ability to maintain higher GSH:GSSG ratios was associated with greater stress tolerance [157]. GSSG accumulation is a key determinant of cell death and growth arrest [158, 159].

Immunolocalization studies revealed that stress-triggered GSSG accumulation occurred in discrete subcellular compartments. Localization studies in Arabidopsis detected little or no accumulation in mitochondria, slight but significant accumulation in the cytosol, and prominent accumulation in vacuole and chloroplasts [156]. GSSG sequestration in metabolically inert vacuole is thought to initiate catabolism, whereas accumulation in chloroplasts could have functional consequences for photosynthetic efficiency. Increased GSSG level is sufficient to trigger protein *S*-glutathionylation [159], which is thought to regulate enzymatic protein activity [160]. A large number of unidentified targets of this posttranslational modification represent chloroplast proteins (e.g., RuBisCO or glucose-6-phosphate dehydrogenase) [160]. However, it is not clear if GSSG accumulation in chloroplast results from import or *in situ* synthesis. Isolated wheat chloroplasts can take up GSSG from the medium [161]. Re-engineering of compartment-specific glutathione synthesis pathways suggested that cytosol-to-chloroplast GSSG transport also occurs *in vivo* [162]. Specific GSSG transporters have been identified in tonoplast but not in the chloroplast envelope [163].

The presence of redox-sensitive cysteines has been shown for mammalian ANXA2 [164] and ANXA1 [165]. They are located in the extended C- or N-terminal end (Cys324 aa for ANXA1, and Cys8 and Cys334 for ANXA2), which confer structural diversity to proteins from this family. It cannot be easily generalized if other annexins contain cysteines susceptible to oxidation. ANXA2 was proposed to directly neutralize H_2_O_2_ with accompanying oxidation of only Cys8 [166, 167]; subsequently, it would be reduced via the NADPH-dependent thioredoxin system (NTS) being thus an ultimate acceptor of electrons from NADPH. Oxidative damage in annexin A2-depleted cells enhanced oxidation of the ANXA2-binding proteins actin and transcription factor JunD. This suggests that ANXA2 can function directly as a protein reductase [168].

The presence of reactive cysteines was confirmed in Arabidopsis annexin ATANN1. Implicated amino acids are localized within the endonexin domains and are highly evolutionarily conserved [20] (Fig C in S1 File). ATANN1 Cys underwent *in planta S*-nitrosylation within 20 min after NO treatment [169] and *S*-glutathionylation within 30 min after ABA induction [23]. MeV treatment resulted in oxidation of both cysteines residues of ATANN1 (Cys111 and Cys 239), although the exact type of modification (mixed disulfide bonds with GSH, or intramolecular disulfide bond) has not been defined [170]. The closest ATANN1 homolog in *Brassica rapa*, BRANN1, appears to form a complex with peroxidase in floral buds [171].

**Fig 11:**
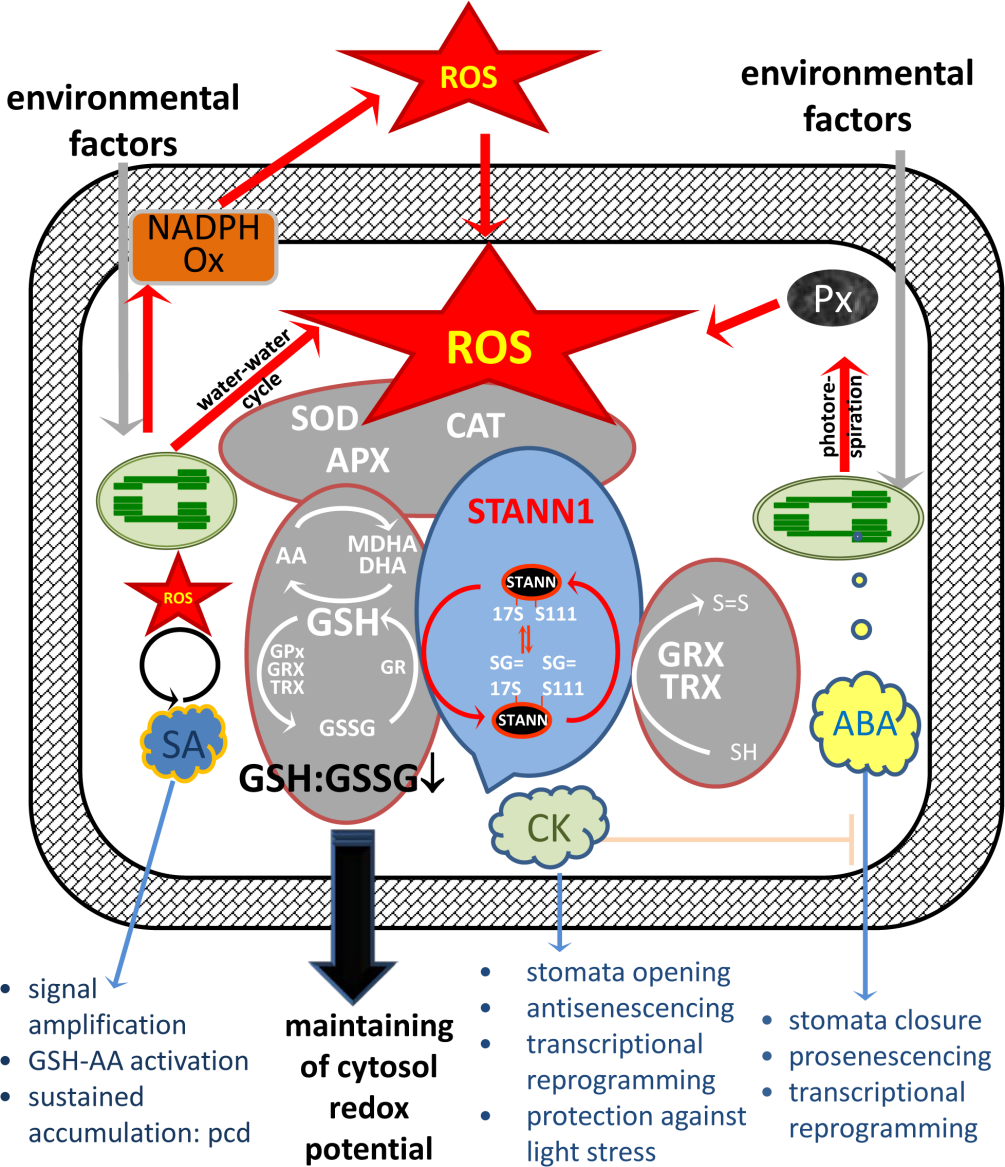
A simplify scheme depicting the interactions between cellular redox state and participation in ROS scavenging mechanisms. Oxidative stress is an unavoidable consequence of environmental stresses. ROS accumulation begins in chloroplasts and then it spreads throughout the whole cell. Activation of a secondary ROS sources e.g. NADPH oxidase complexes or photorespiration resulted in substantial H_2_O_2_ accumulation in cytosol. To avoid deleterious effects of ROS several compartment-specific mechanisms evolved, including accumulation of low-molecular-weight antioxidants (glutathione, ascorbate), scavenging enzymes (catalases CAT, ascorbate peroxidases APX, and sodium dismutases SOD) and protein thiols (peroxiredoxins PRX, glutaredoxins GRX, and thioredoxins TRX) that undergoes a reversible cycles the thiol-disulphide exchange. The redox-sensitive proteins sense, transduce, and translate ROS signals into appropriate cellular responses. Thus, precise regulation of size and redox status of the thiol pool is of essential importance for induction of appropriate responses. In plant cells glutathione is present in different compartments in milimolar concentrations and in quiescence it maintained largely in reduced state due to activity of glutathione reductases (GR) at expense of NADPH. Stress-induced ROS accumulation stimulates oxidation of glutathione (GSSG) and in the same time *de novo* synthesis of GSH. Disturbances in GSH/GSSG ratio might non-specifically influence several downstream pathways, e.g. by induction of thiol-disulfide exchange on target proteins. Cellular redox potential depends primarily on the total concentration of the total glutathione and the extend of its oxidation. GSSG accumulation did not disturb the redox potential if it is compensated by increasing the total glutathione concentration. However, if size of total pool remains unchanged when the GSH:GSSG ratio increased the cell redox potential in the cytosol become more positive. We propose that the improved stress tolerance of annexin STANN1-overexpressing potato plants results from amelioration of oxidative shift of the cytosolic glutathione redox potential. Elevation of STANN1 level had a pleiotropic effect on plant metabolism and physiology what suggested that not one specific but several downstream signaling pathways were touched. Disruption of the glutathione redox potential is sufficient to induce such effect; e.g., in transgenic tobacco with constitutive up-regulation of glutathione content MAPK and SA signaling pathways were modified. Annexin posses oxidation-sensitive cysteines and can act as a reductant influencing thus the redox potential. During stress in transgenic plants the capacity of cytosol redox buffer was more reducing compared to WT what prevents oxidation of downstream targets and modulate timing as well as magnitude of stress response. It had a beneficial effect on cell survival, photosynthesis and delay senescence. Similar effects were observed in tobacco and Arabidopsis plants and over-expressing particular elements of antioxidant systems.

STANN1 contains two cysteine residues, Cys17 and Cys111. Among potato annexins, the former is unique for STANN1 and STANN3.2, whereas the latter is homologous to Arabidopsis Cys111. This arrangement resembles that of ANXA1, with reactive Cys8 in the N-terminal amino acid. Elevated STANN1 levels mitigated photooxidative stress and diminished ROS accumulation, which suggested that STANN1 enhanced the capacity of cytosolic antioxidant buffer. Therefore, it appears that the evolutionarily conserved cysteine homologous to ATANN1 Cys239 is not necessary for such activity. Plant annexins can prevent ROS over-accumulation in a similar way to that previously shown for ANXA2: by direct ROS neutralization and further regeneration by NADPH-dependent thioredoxin/glutaredoxin system. Therefore, annexin would function as an ultimate acceptor of excess electrons leaking from over-reduced PETC. Alternatively, STANN1 may be used as an acceptor for ROS diminishing thus GSSG formation (Fig 11). Annexins are abundant cytosolic proteins (accounting for up to 2% of the total soluble proteins). They possess redox-sensitive cysteines and could participate significantly in the cellular protein thiol pool. In transgenic plants overexpressing STANN1, annexin levels are higher and the antioxidant protective effect is increased. Reduced GSSG accumulation prevents a decline in the GSH:GSSG ratio and over-oxidation of the cellular environment. The latter mechanism could explain the broad-specificity of annexin-mediated protection, which is functional in bacteria and photosynthetic and non-photosynthetic eukaryotic cells. Glutathione is one of the most abundant non-protein thiols; it is present in cyanobacteria and proteobacteria, and in all mitochondria- or chloroplast-containing eukaryotes [172, 173]. The mechanism of GSH-mediated regulation and maintenance of cellular redox status is similar in all living organisms. Reversible oxidative thiol modifications modulate the function of proteins involved in many different pathways, including gene transcription, translation, protein folding, metabolism, signal transduction, and apoptosis.

## Conclusions

The results obtained in this study clearly indicate that annexin overexpression has potential application for developing drought-tolerant crops. Enhanced drought tolerance in transgenic potato overexpressing STANN1 confers greater tolerance to high light stresses, stomatal closure, and diminished CO_2_ supply. ROS accumulation was attenuated, which improved chloroplast function; genetically modified plants maintained efficient PSII under stress conditions. Maintenance of a high photosynthetic yield under sub-optimal conditions had a beneficial effect on crop yields and biomass production. Annexins are a promising target for manipulation of plant tolerance to environmental conditions.

## Supporting Information

S1 File This File contains Figures A-G. (PDF)

S2 File This File contains Tables A-D. (PDF)
