# Supplementary material for: Potato Annexin STANN1 Promotes Drought Tolerance and Mitigates Light Stress in Transgenic Solanum tuberosum L. Plants
Source: PLoS One. 2015 Jul 14;10(7):e0132683. doi: 10.1371/journal.pone.0132683 (PMC4501783; doi:10.1371/journal.pone.0132683)
Supplement: S1 File — (PDF) [file pone.0132683.s001.pdf]

Figure A. Construction of transgenic plants.

(A)

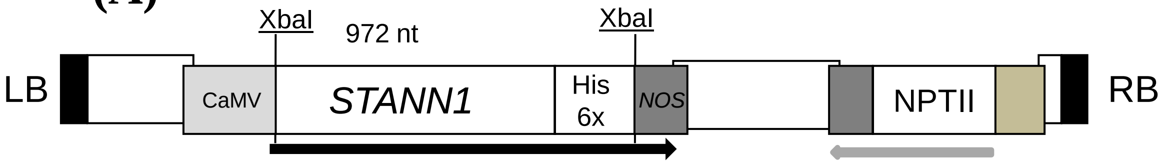

(B)

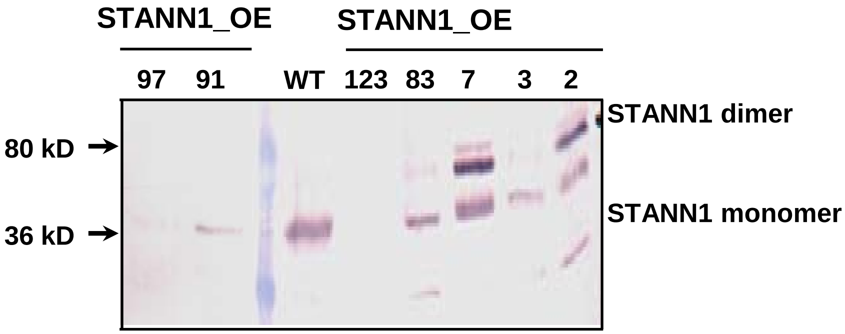

(A) Structure of the T-DNA region from pROK2 carrying STANN1_His6x that was used for *Agrobacterium* -mediated transformation. LB – left border; RB – right border; NPTII – neomycin phosphotransferase II, CaMV – cauliflower mosaic virus 35S promoter; NOS – nopaline synthase terminator;

(B) Expression of STANN1_His6x protein in F1 transgenic potato lines. Proteins were isolated from leaves of WT and F1 transgenic lines S-2, S-3, S-7, S-83, S-91, S-97 and S-123 grown *in vitro*. His-tagged proteins were purified with Ni-NTA agarose, subjected to SDS_PAGE and blotting followed by detection with anti-His primary Ab. The band detected in WT represents *Arabidopsis* annexin ATANN1_His6x (molecular weight *ca* 36 kD) produced in *Escherichia coli* that was added before purification to the ground protein to STANN1_His6x easily dimerized hence the two bands were detected, the lower with molecular weight corresponding to monomer and the upper corresponding to dimer.

Figure B. Schematic characteristic of experimental drought.

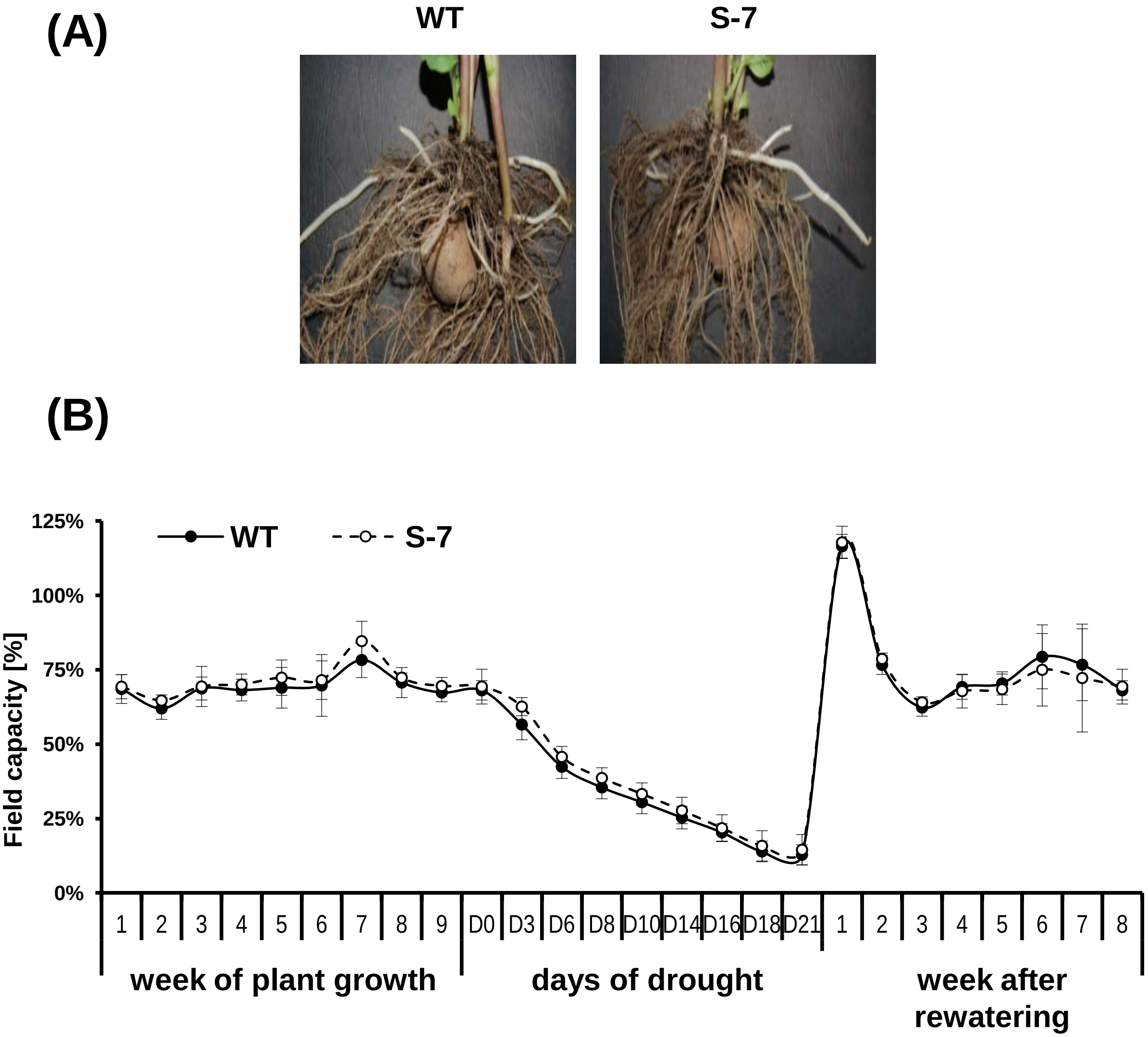

(A) Potato WT and S-7 plants after 8 week of growth at the phase of experimental drought implementation. Transgenesis has no impact on tuber development. Formation of stolon hooks and stolon swelling as well as first tubers are visible.

(B) Field capacity (FC) was normalized at the beginning of experiment and maintained at constant level (app. 65%); for control (well-irrigated plants) FC was maintained at this level throughout the whole experiment. For experimental drought FC was gradually lowered to 20% and kept at this level un till the end of drought. Rewatering was applied by full water saturation of the soil and after gravity draining of excess water FC was kept at the 65% untill the end of experiment.

Figure C. Multiple alignment of amino acid sequences of putative annexins from potato and selected annexins from human, Arabidopsis and cotton.

```
                |---------------------------Domain 1------------------------------------------|
ANXA5     MAQVLRGTVTDFPGFDERADAETLRKAMKG----LGT EESILTLLTSRSNAQRQEISAAFKTLFGRD------LLDDLKSELTGKF---EKLIVAL 85
GHANN1    MASL-T-VPAHVPSAAE—-DCEQLRSAFKG----WGTNEKLIISILAHRTAAQRKLIRQTYAETFGED------LLKELDRELTHDF---EKLVLVW 81
ATANN1    MATL-K-VSDSVPAPSD--DAEQLRTAFEG----WGTNEDLIISILAHRSAEQRKVIRQAYHETYGED------LLKTLDKELSNDF---ERAILLW 81
STANN1    MASL-T-VPAEVPSVAE--DCEQLRSAFKG----WGTNEKLIISILAHRNAAQRKLIRQTYAETFGED------LLKELDRELTHDF---EKLVLIW 81
STANN2    MESL-K-IPELVPSAAE--DSQQLRKAFVG----WGTDEACIIKILGHRNAAQRKLIRETYEATYEKD------LLQDLDGEISGDF---QRVVHLW 81
STANN3.1  MATL-R-IPDVVPSPTE--DSETLMKSFKG----LGTNEKSVISVLGHRNASQRKKIRETYQQLYNRS------LVDDIFSELSGDF---KKAVVLW 81
STANN3.2  MGTL-R-IPDVVPSPDE--DCETLMKSFKG----LGTNEKSVISVLGQRNASQRKKIRETYQQLYNKS------LIDDINSELSGDF---RKAVILW 81
STANN3.3  MGSL-L-VPDFIPSPIQ--DAETLRKSFKGHFL LGT EKAIITVLGHRDESQRKKIKEAYQQLYNKS------LIDDLHSELSGDF---RKAVILW 85
STANN4    MAEA-N-------SYAA------LTKAFSG----LGVDENLFISTLGKWNRHQRESYRVSTPGFFREDERQFQRWDDQHILQLRQEFLRLKDAVVLY 80
STANN5    MATL-S-IPPVLTTPRD--DAMHLYKAFKG----FGCDKAAVINILAHRDATQRALIQQEYRTMYSED------LTKRLVKELSGKL---EKAILLW 81
STANN8    MSTI-IY--PENTSPVA--DAEAIRKACQG----WGT EKAIISIFGHRNATQKKLIRQAYEELYSED------LVKRLESELSGQF---EKAVYRW 80
STANN9    MSSL-K-VPASVPDPYE--DAEQLKKAFKG----WGTNEDLIIQILAHRNAAQRKLIRDSYAAAYGED------LLKDLDSELSSDF---QRVVLLW 82

           |------------------------Domain 2-----------------------------------|
ANXA5     MKPSRLYDAYELKHALKGAG---TNEKVLTEIIASRTPEELRAIKQVYEEEYGSSLEDDVVGDTSGYYQRMLVVLLQANRDPDAG          164
GHANN1    TLDPSERDAHLAKEATKRWT---KSNFVLVELACTRSPKELVLAREAYHARYKKSLEEDVAYHTTGDHRKLLVPLVSSYRYGGEE          159
ATANN1    TLEPGERDALLANEATKRWT---SSNQVLMEVACTRTSTQLLHARQAYHARYKKSLEEDVAHHTTGDFRKLLVSLVTSYRYEGDE          159
STANN1    TLDPSERDAYLAKEATKRWT---KSNFVLVEIACTRSPKELVLAREAYHARNKKSLEEDVAYHTTGDHRKLLVPLVSSYRYGGDE          159
STANN2    TMAPAERDACLANEAIKHLP---GSNCIIMEIACARSSVDLFKVRQNYQARYKKSLEEDVADHSTGDFRKLLVSLVTALRYEGEE          159
STANN3.1  TYEPSERDARLANEALKSKKKTITQLQVIVEIACASSPDHLVAVRQTYCGLFNCSLEEDIAANVPMPVQKVLIGLVRSYRYDKEL          161
STANN3.2  TYEPSERDARLANGALKSRKKTITQLQVIVEIACASSPDHLVAVRQAYCGLFNRSLEEDITANVPMPIQKILIGLVRSYRYDKEL          162
STANN3.3  THDPPERDARLANEVLNSWIHDVTRLQVIVEIACASTPDHLVSVRQTYCALFGCSLEEDIIAHVSLPVQKVLISLVSSYRYNKEL          162
STANN4    TMHPWERDARLFKEALLCKY---LNMMFSLRLLALDPLKIFWEPEKLTIPFLNTQLRKTLPSIFEVPKRKLLVALVSSYRYEGPR          159
STANN5    MYDPAGRDAILVRQALSADS---IDLRAATEVICSRTPSQIVHLKQLYHSMNGIYLEHDIALHASDDHKKLLLAYVTTMRYEGPE          160
STANN8    ILNPRDRDAVILHVAIKERA--IPNYRVVVEYSCIYSPEELLAVKRAYQARYKTSVEEDIAQYSTGHLRKFLVGLVGTYRYVGDE          163
STANN9    TLSPAERDAYLVNEATKRLT---ASNWVIMETACTRSSDDLFKARQAYHARYKKSLEEDVAYHTSGDFRKLLVPLITAFRYEGDE          160

              |--------------------------Domain 3--------------------------------------------|
ANXA5     IDEAQVEQDAQALFQAGE-----LKWGTDEEKFITIFGTRSVSHLRKVFDKYMTISGFQIEETIDRET-SGNLEQLLLA---VVKSIRSIPA   246
GHANN1    VDLRLAKAESK-ILHEKI-----SDKAYSDDEVIRILATRSKAQLNATLNHYKDEHGEDILKQLEDGD---EFVALLRA---TIKGLVYPEH   239
ATANN1    VNMTLAKQEAK-LVHEKI-----KDKHYNDEDVIRILSTRSKAQINATFNRYQDDHGEEILKSLEEGDDDDKFLALLRS---TIQCLTRPEL   242
STANN1    VDLRLAKAESK-VLHEKI-----SDKAYSDDEVIRILATRSKAQLNATLNHYKDEYGEDILKQLEDED---EFVALLRA---TIKGLVYPEH   239
STANN2    VNMELASDEAK-ILHEKI-----SDKADSDEEFIRILSIRSKTQLNATFNQYNDKFGNAINKDLRANP-KDQYLTLLRS---AIKCLMEPEK   241
STANN3.1  VDPSFANEESA-ILRETI-----RTKQLDSDNFLLILSTRNVHQLRATFECYKQNYGFSIDQDMKSCG-KGLLESILKV---VIWCIDSPEK   243
STANN3.2  VDPSTANEEAA-ILREAI-----RTKQLDHDNFLFILSTRSSCQLRATFECYKQNYGFSIDQDIKSCG-KGLLESILKV---VIWCIDSPEK   244
STANN3.3  VDHSTANLEAS-KLREAT-----RTKQLDSDELVLILSTRNIHQLKATFECYKQNYGFSIDQDITNCG-EGLLESILKV---VIWCINSPEK   245
STANN4    VSDDLAKSEAK-IFVNAIKNANKKKLIDEEEEIVRILSTRSKLHLKAIYSHYKEITGNFLDEDLEGDL-------TMKQ---VVQCLCVPKA   230
STANN5    VDRASVDHDAKALYKAGE-----KKLGTDEKTFIRIFSERSRAHLAAVSSAYHSMYSRKLKKAVKSET-SGLFEFALLT---ILQCAENPAT   242
STANN8    INARVANSEAD-ILHNAI-----SNKEFNNEEIVRIICTRSTTQLVATLNRYKDYYGSSIIKHLIDDTNDEDYKEYLLALRTTIRCINDPQK   249
STANN9    VNMTLARKEAN-ILHEKI-----SDKAYNDEEIIRIISTRSKAQLSATFNHYNDHHGHEIIKDLEADDDDDEYLKLLRA---AIECLKTPEK   244

          |-------------------------------DOMAIN 4---------------------------|
ANXA5     YLAETLYYAMKGAGTDDHT---LIRVMVSRSEID-LFNIRKEFRKNFATSLYSMIKGDTSGDYKKALLLLCGEDD          320
GHANN1    YFVEVLRDAINRRGTEEDH---LTRVIATRAEVD-LKIIADEYQKRDSIPLGRAIAKDTRGDYESMLLALLGQEED--   314
ATANN1    YFVDVLRSAINKTGTDEGA---LTRIVTTRAEID-LKVIGEEYQRRNSIPLEKAITKDTRGDYEKMLVALLGEDDA--   317
STANN1    YFVEVLRDAINRRGTEEDH---LSRVIATRAEVD-LKTIANEYQKRDSIPLGRAIAKDTGGDYENMLVALLGQEEE--   314
STANN2    YFEKVLRLAMKGFGTDEES---LTRVVATRAEVD-MELIKEKYYKRNSVTLESAISDDTSGDYEKMLLALIGHGNL--   316
STANN3.1  HFAEVVRASIVGIGTDENS---LTRAIVTRAEVD-MMKVRGEYFIANKTNLDSAVIGDTSGDYMKFLMTLLGAK----   317
STANN3.2  HFAEVVRASIVGFGTDEDS---LTRAIVTRAEVD-MMKVRGEYIIANKTSLDSAVIGDTS-----------------   303
STANN3.3  HFAEVVKVSTDGLGTNEDS---LSRAIVTRAEID-MIKVKEEYLKMKDTALEYAVADDTSGHYREFLMTLLGANDSSL   325
STANN4    YFSKILIASLR-L[illegible]DESAKDSVTRVIVTRADDDDMKQIKEEFQSKYGTTLAAKIAEVANGSYKDFLLTIIAKSD---   316
STANN5    YFAKELHKAMKGLGTNDTT---LIRIVATRTEID-MQYIKAEYQKKHKKSLNDAVHSETSGDYRTFLLISSGACS---   316
STANN8    YYEKVIRYAINESGTDEES---LTRVIVTRAEKD-LKEIKELYYKRNSVTLDHAITNHTCGNYKAFLLTLLGNEN---   319
STANN9    YFEKVLRLAIKKLGTDEWD---LTRVVATRAEVD-MERIKEEYHRRNSVTLDRAIAADTSGDYEKMLLALIGHGDA--
          317
```

The alignment was done with Cobalt (Constrain-based Multiple Alignment Tool). Gene Bank Acc Nos of sequences are: human AnxA5 (NP_001145.1), cotton (*Gossypium hirsutum)* GHANN1 (1N00), *Arabidopsis thaliana* ATANN1 (2Q4C), *Solanum tuberosum* annexins: STANN1 (PGSC0003DMG4000177114), STANN2 (PGSC0003DMG40002817), STANN3.1 (PGSC0003DMG4000221817), STANN3.2 (PGSC0003DMG401019427), STANN3.3 (PGSC0003DMG402019427), STANN4 (PGSC0003DMG400019446), STANN5 (PGSC0003DMG400007966), STANN8 (PGSC0003DMG400007482) and STANN9 (PGSC0003DMG40001879). Boundaries of endonexin repeats (orange rectangles) were determined on the basis of crystallized plant annexins from cotton, GHANN1 and Arabidopsis ATANN1. Functional amino acid motifs (either predicted or previously indicated for plant annexins) are highlighted. Conserved histidine 40 residue is in red; methionine and cysteines from C3 cluster are in blue and underlined. Calcium binding motifs G-X-GTD-{38-40}-D/E are in green boxes; potential N-terminal acylation motif is in bold; putative actin-bindin domains IRI are bolded in yellow boxes. C-terminal peptide similar to 14-3-3 proteins is marked by blue rectangle. Amino acid residues of high conservation are shown in red, medium - in blue.

Figure D. Drought tolerant phenotype of transgenic S-7 potato plants.

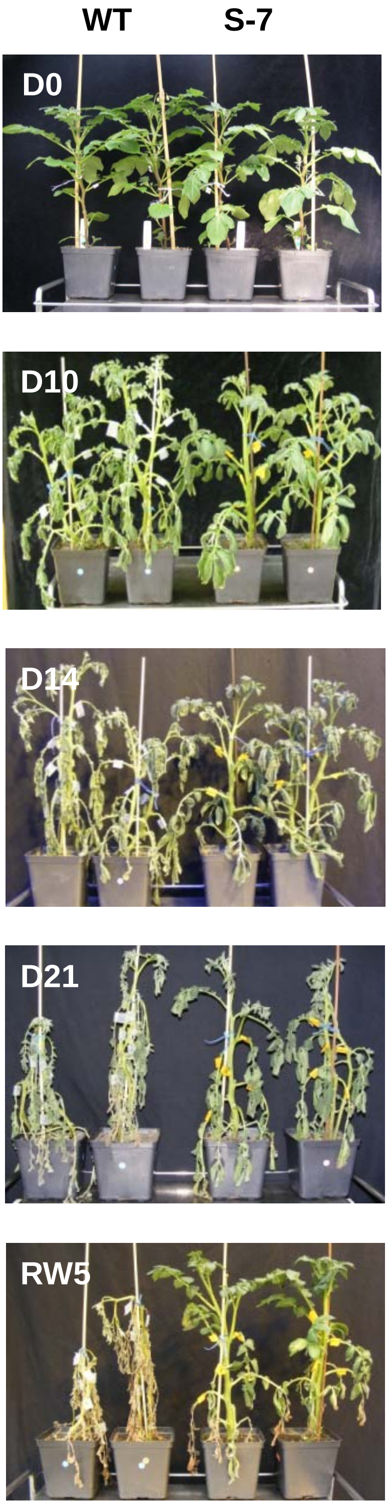

Each image depicts two WT plants (left side) and two transgenic S-7 plants (right side) subjected to experimental drought. Drought was started on D0 and lasted 21 days. During that time watering was gradually reduced so as to lower the FC to 20%. After reaching that level it was maintained until 21 days after onset of experiment. The soil was than fully saturated with water (rewatering) and FC was maintained at 65% until the end of experiment. D10 - irrigation withheld for 10 days, D14 - irrigation withheld for 14 days, D21 - irrigation withheld for 21 days, RW5 – rewatered for 5 days. Experiments were repeated four times and similar results were obtained both in greenhouse and in growth chamber.

In WT symptoms of wilting clearly appeared after 10 days of drought; in S-7 they were apparent only after 2 weeks. On the 21st day WT were severely affected with damaged stems and dry leaves. At the same time in S-7 plants the upper leaves still maintained turgor. After rewatering only a few leaves in WT regenerated; instead, new shoots developed from below-ground parts after at least a week of regular irrigation. In contrast, the S-7 plants preserved their upper leaves and after rewatering returned to a normal healthy look within hours. The exact number of irreversibly damaged leaves varied between experiments, but it was always significantly lower than in WT.

Figure E. Potato yield during drought.

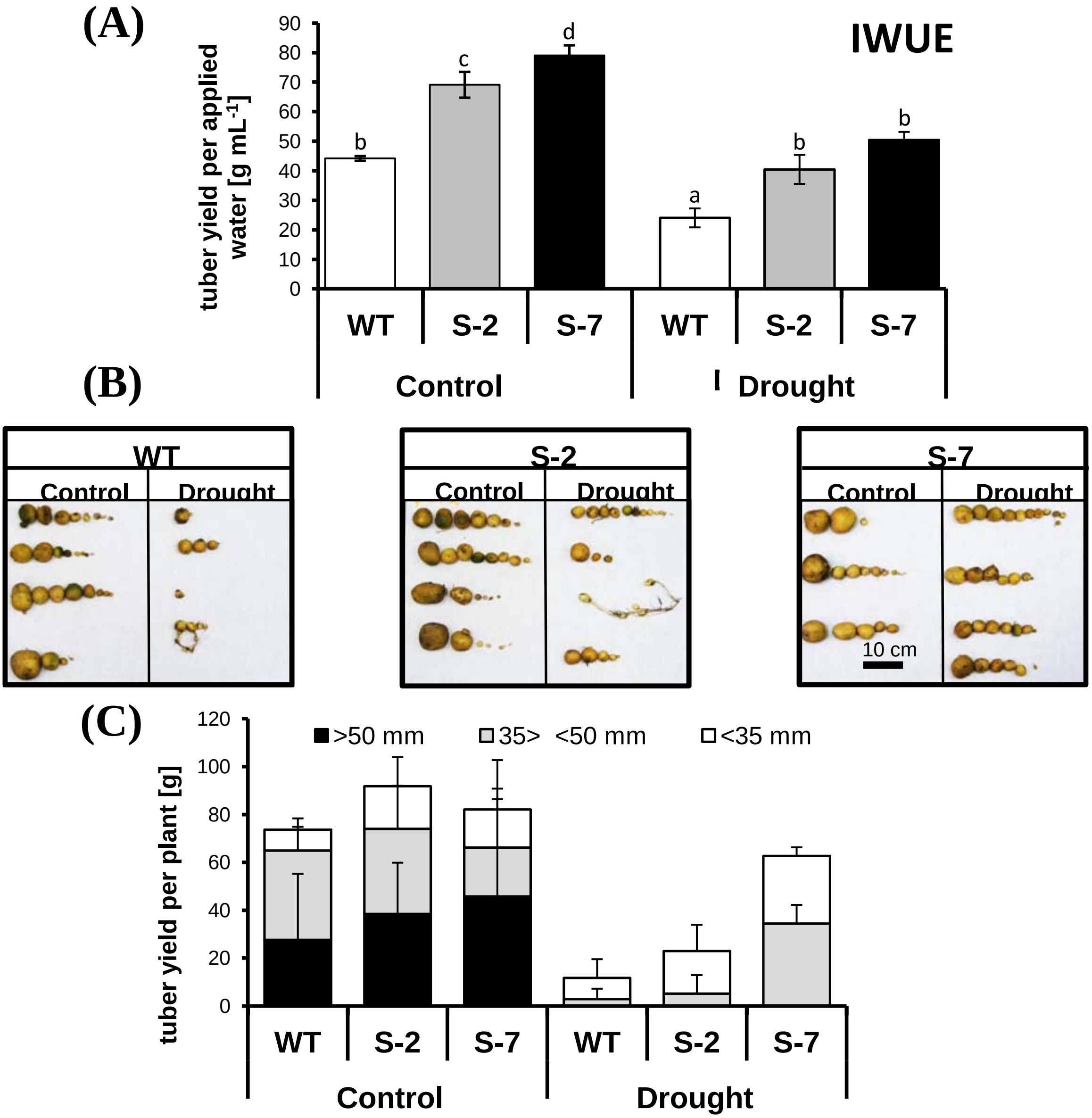

(A) Irrigated Water Use Efficiency (IWUE) is a quotient of crop produced per unit per amount of water supplied (IWUE = Y / W [g/pot/mL of water])

(B) An exemplary tuber yield per plant. Potato plants WT, S-2, and S-7 were grown in a greenhouse. After 8-10 weeks of growth plants were subjected to drought stress by restricting irrigation to achieve 20% FC and kept at this level until 14th day. After that time plants were rewatered and cultivated in optimal conditions for additional 10 weeks until physiological maturity. Tubers were lifted immediately after withering of haulms. The weight of all fresh tubers from single plant was determined immediately after harvesting. Experiments were repeated twice and gave similar results.

(C) Quantification of tuber yield experiments. Results are shown as mean SD (*n*=10)

Figure F. Expression of genes coding for PSII proteins and HSPs.

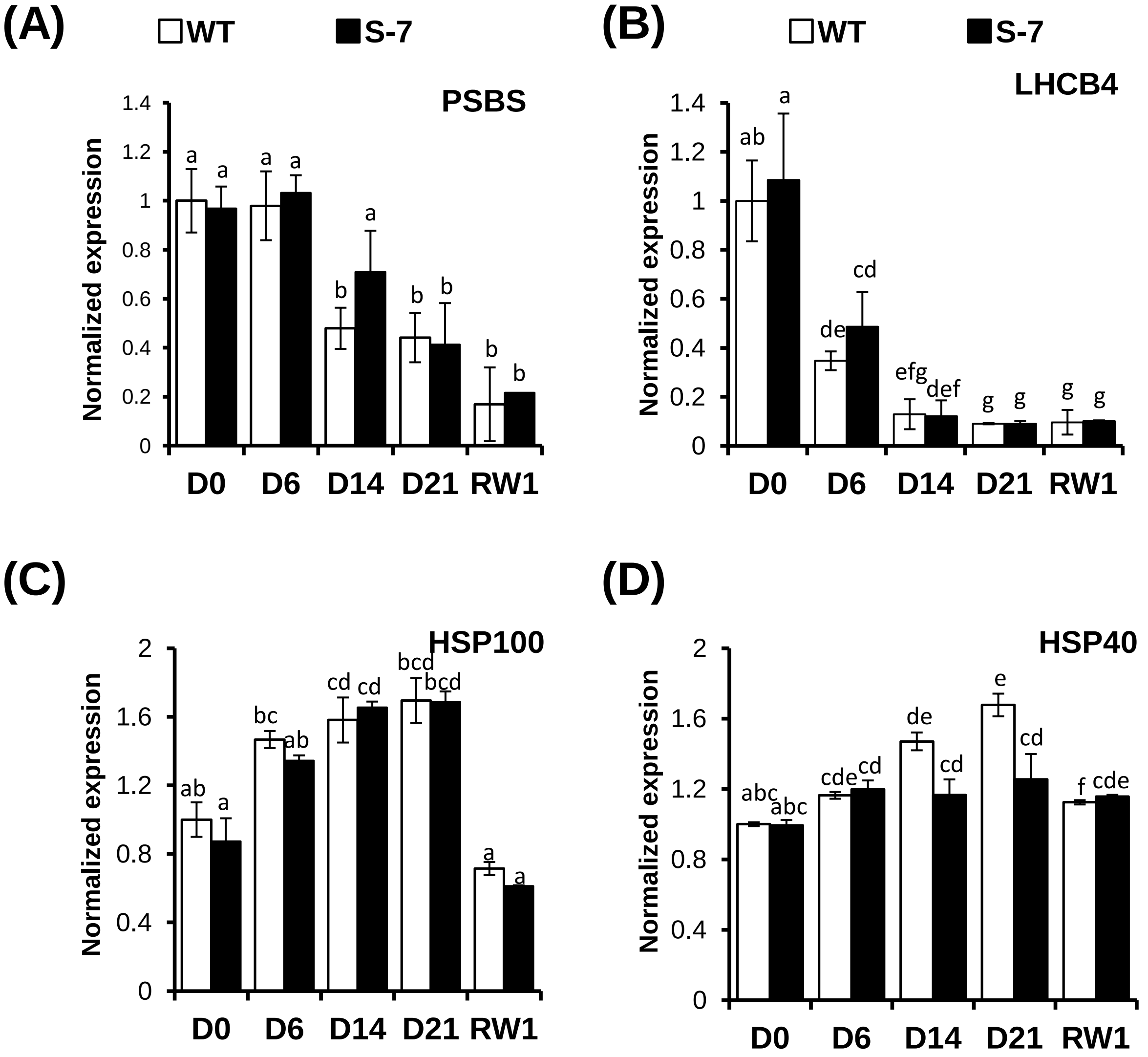

Relative quantification of *PSBS* (A), *LHCB4* (B) *HSP100* (C) and *HSP40* (D) mRNAs in leaves of WT (white bars) and transgenic S-7 (black bars) potato plants during three-week drought and after rewatering. The data represents the mean SE from at least four measurements. Homogenic groups are determined by Tukey HSD (Honestly Significant Differences) test, the same letters designate days which are not significantly different at $P < 0.05$ and belong to the same homogenic group.

Figure G. The effect of photooxidative stress on potato leaves.

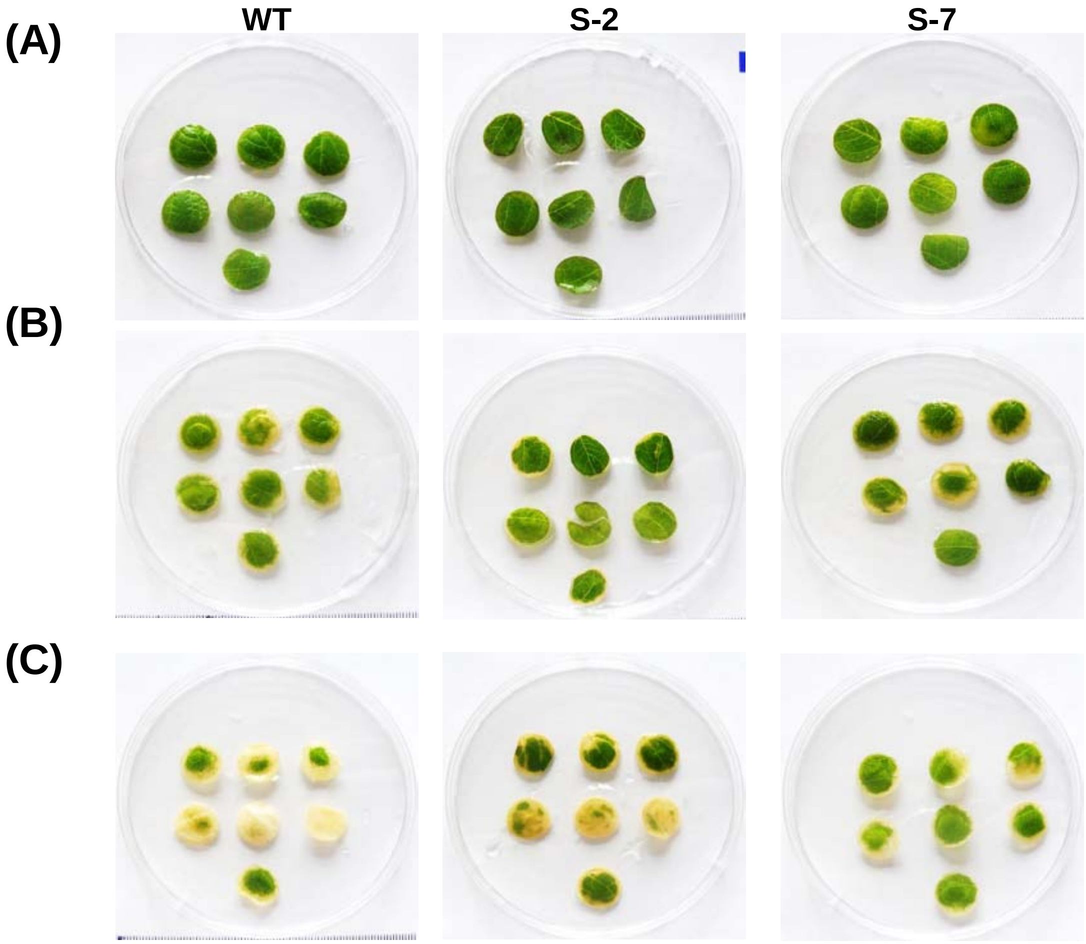

Leaf discs (Φ ~ 1 cm) were excised from leaves of WT or transgenic plants S-2 and S-7 and immediately infiltrated with (A) 50 mM Tris, pH 7.5 (B)10 μm MeV or (C) 50 μM MeV. Subsequently, leaf discs were exposed to light of 150 PPFD for 30 h.
