# Supplementary material for: Potato Annexin STANN1 Promotes Drought Tolerance and Mitigates Light Stress in Transgenic Solanum tuberosum L. Plants
Source: PLoS One. 2015 Jul 14;10(7):e0132683. doi: 10.1371/journal.pone.0132683 (PMC4501783; doi:10.1371/journal.pone.0132683)
Supplement: S2 File — (PDF) [file pone.0132683.s002.pdf]

Table A. Primer pairs used for identification of potato annexins.

**Gene ID** (PGSC0003)	**Gene symbol**		**Primers for CDS isolation 5'-3'**	**Gene lenght [bp]**	**No of exons**	**CDS [bp]**
DMG400017714	*STANN1*	F	ATGGCAAGTCTTACAGTTCC	2402	5	942
		R	TTCCTCCTCTTGTCCAAGTAAAGCCA			
DMG400021817	*STANN2*	F	ATGGATCTAGGATTTGAACTT	2737	4	1191
		R	AAGATTTCCATGGCCAATTAAGGCTAAAAGCA			
DMG402019427	*STANN3.1*	F	ATGGGCAGTCTCTTAGTACCA	2725	6	954
		R	CAAACTACTATCATTGGCACCAAGCAGG			
DMG401019427	*STANN3.2*	F	ATGGGTACACTGAGAATCCCA	2957	6	912
		R	TGATGTGTCACCAATAACTGCACTATCAAG			
DMG400040554	*STANN3.3*	F	ATGGCTACACTGAGAATCCC	3148	6	975
		R	TTTGGCACCAAGCAGTGTCATGAGGAA			
DMG400019446	*STANN4*	F	ATGGCCGAGGCTAATTCGTATG	1933	6	948
		R	GTCAGATTTTGCAATTATAGTAAGCAAGAAAT			
DMG400007966	*STANN5*	F	ATGGCTACTCTGAGTATTCCTCC	4814	6	948
		R	GTGAGCAGGCCCCAGAAGAGATAGA			
DMG400007482	*STANN8*	F	ATGTCTACCATCATTTACCCGG	1547	6	957
		R	ATTTTCATTTCCCAAAAGAGTTAGGAGGAAAG			
DMG400001879	*STANN9*	F	ATGTCTAGTCTTAAAGTTCCAGCATCA	3469	5	951
		R	AGCATCTCCGTGCCCAATCAAAGCCA			

Primer pairs corresponding to the predicted 5' (F) and 3' (R) ends of the particular annexin genes were designed on the basis of published potato genome sequence. Gene length refers to the total length of exons and introns. Individual primer pairs (F - forward, R - reverse) were designed with PrimerSelect, Laser Gene10.0 DNASTAR (USA).

Table B. Primer pairs used for sq-RT-PCR.

Gene symbol		Primers sequence 5'-3'	Ta [^{o}C]	PCR product length [bp]
STANN1	F<br>R	TTAGCCACAAGGAGCAAAGC<br>TCCCTCTTCTGGTACTCGTTAG	57	366
STANN2	F<br>R	GGCTTATAGTGATGAGGAGTTC<br>AAGATTTCCATGGCCAATTAAGGCTAAAAGCA	57	402
STANN3.1	F<br>R	CTCCATTGACCAGGACATGAAGAG<br>TTTGGCACCAAGCAGTGTCATGAG	58	300
STANN3.2	F<br>R	CTGCTATCCTTCGTGAAGCCATAC<br>GGCTCTCGTTAGAGAATCCTCATC	58	385
STANN3.3	F<br>R	CGAATTGTGGCGAAGGTC<br>AGCAGGGTCATCAGGAAC	54	292
STANN4	F<br>R	AGCTCCATCTTAAGGCCATC<br>GTAACTATGACTCGGGTCACTG	58	343
STANN5	F<br>R	GGAGAAGAACTGGGAACTG<br>GCGTGGCTACTATCCTAATG	59	412
STANN8	F<br>R	GTACAAGGAGCACCACTCAAC<br>CTCAGCTCGCGTAACTATCAC	58	377
STANN9	F<br>R	GTGCAACGTTCAACCACTAC<br>CCCAATCAAAGCCAGAAG	58	344
HSP100	F<br>R	GGGAGTACAAGAAAGAGTATGGTGA<br>CTGCACCAACAACAGTATGTATCTC	60	575
HSP40	F<br>R	CCTTCCAAAAGATCCGTCAA<br>TTACGAAAGGGACTCGCCTA	52	228
PSBS	F<br>R	GCTCCTCCCAAAAAGGTTGCACCA<br>GGCCAGTAGCAGGGGAAGGGT	60	322
LHCB4	F<br>R	AAGACGCCGGAAAGGTTGA<br>TTAAGAGAAGAATCCGAAGGTGTC	53	380
EF1A	F<br>R	TCACATCAACATTGTGGTTATTGG<br>TTAAGCTGGTCAAGAGAGCCTCAAG	55	350

Primers for semi-quantitative analysis of expression of annexins and other genes in potato. Individual primer pairs (F- forward, R- reverse) were designed with PrimerSelect, Laser Gene10.0 DNASTAR (USA) to span intron–exon boundaries to exclude interference from genomic DNA contamination. Amplified fragments were between 300 and 500 base pairs. The genes were selected from PGSC_DM_v3.4_pep_fasta containing database of potato virtual translation products on the basis of their homology to annotated Arabidopsis genes. Analyzed genes were as follows: annexins: *STANN1-9; HSP100* (heat shock protein 100 kDa); *HSP40* (heat shock protein 40kDa, DNAJ); *PSBS* (chlorophyll a/b- binding photosystem II 22kD subunit S); *LHCB4* (light-harvesting complex binding protein 4). As a reference the housekeeping gene for Elongation Factor a1 (*EF1a*) was used.

Table C. Characterization of putative potato annexin proteins.

Annotated CDS	Protein symbol	AA	M_W [kD]	pI	Anx repeats	Localization predicted by WoLF PSORT
DMT400045665	STANN1	314	35.80	5.37	4	cyto_ER, vacu, chlo, nucl
DMT400056154	STANN2	315	35.90	5.30	4	cyto, cysk, chlo, nucl, plas
DMT400049998	STANN3.1	317	35.61	6.75	4	nucl, cyto, chlo
DMT400049997	STANN3.2	303	34.01	7.19	4	nucl, chlo, cyto
DMT400090983	STANN3.3	325	36.61	5.35	4	cyto, nucl, cysk, chlo
DMT400050067	STANN4	316	36.62	8.42	4	cyto, cyto_ER, nucl, cysk
DMT400020562	STANN5	316	35.53	8.95	4	chlo, cyto, nucl, mito, plas, extr
DMT400019344	STANN8	309	36.85	6.48	3	cyto, cysk
DMT400004741	ANNST9	311	36.32	5.42	4	cyto, chlo, nucl, plas, cysk

chlo – chloroplast; cyto – cytoplasm; cyto_ER – cytoplasm/membrane of endoplasmatic reticulum; cysk – cytoskeleton; ER – endoplasmatic reticulum; extr – extracellular; mito – mitochondria; nucl – nucleus; plas – plastids; vacu – vacuole;

Table D. Cytokinins in leaves of WT and S-7 potato plants under drought.

	Days of drought							
[pmol g^{-1} FW]	D0		D6		D14		RW1	
	WT	S-7	WT	S-7	WT	S-7	WT	S-7
**tZR**	1.52±0.28	1.56±1.26	3.28±0.51	2.36±0.32	0.29±0.06	0.26±0.12	0.93±0.34	0.64±0.13
**tZ**	1.42±0.06	1.56±0.58	2.26±0.50	1.27±0.02	1.44±0.4	1.35±0.05	1.33±0.63	2.39±1.08
**iPR**	3.16±2.04	1.56±0.2	4.48±0.27	3.17±1.95	2.91±0.66	4.66±1.02	3.79±1.75	6.98±0.72
**iP**	0.21±0.15	1.56±0.03	0.30±0.06	0.27±0.09	0.75±0.05	0.69±0.17	0.75±0.51	1.37±0.23
**cZR**	0.36±0.00	1.56±0.01	0.38±0.03	0.32±0.10	0.82±0.05	0.79±0.18	0.55±0.04	0.51±0.35
**cZ**	0.14±0.06	1.56±0.16	0.49±0.09	0.54±0.09	3.63±0.36	4.95±0.91	1.01±0.18	1.95±0.33

S. tuberosum WT and transgenic S-7 plants were subjected to 2-week drought or well-watered. At time points indicated 0.5 g of tissue (without the main vein) was collected 4 hours after beginning of the day from fully expanded leaves. Hormone levels were analyzed by LC-MS as described in Materials and Methods (n=3). Data are shown as pmol g^{-1} FW.
Abbreviations: tZR, *trans*-zeatine riboside; tZ, *trans*-zeatin; iPR, isopentenyl adenosine riboside; iP, isopentenyl adenine; cZR, *cis*-zeatin riboside; cZ, *cis*-zeatin.
